# Phenotypic variation in growth and biofilm formation of *Leuconostoc* spp. from sugar beet factories

**DOI:** 10.3389/fmicb.2025.1745936

**Published:** 2026-01-15

**Authors:** Sanjay Joshi, Gillian O. Bruni, Tia Zimmerman, Evan Terrell, Johnathan Salter, Md Nayeem Hasan Kashem, Sunghyun Nam

**Affiliations:** 1 U.S. Department of Agriculture, Agricultural Research Service, Southern Regional Research Center, New Orleans, LA, United States; 2 U.S. Department of Energy, Oak Ridge Institute for Science and Education, Oak Ridge, TN, United States

**Keywords:** biofilm, bioreactor, dextran, exopolysaccharides, *Leuconostoc*, sucrose

## Abstract

*Leuconostoc* bacteria are common colonizers of sugar crop processing environments, resulting in sucrose losses and the formation of exopolysaccharides (EPS) and biofilms that can lead to reduced product quality and higher operational costs. Although *Leuconostoc* species are present in abundance, strain-specific differences in biofilm formation, EPS production, and matrix structure are not well understood. In this study, nine sugar beet factory-derived *Leuconostoc* isolates were grown and evaluated using a combination of batch adherence and continuous flow biofilm bioreactor assays, cryo scanning electron microscopy (SEM), EPS quantification, viscosity testing, and growth rate analysis to determine which phenotypes correlate with biofilm formation. The results from the adherence batch-phase biofilms indicated significant phenotypic variation among isolates, with the highest bacterial proliferation by *L. suionicum* BSDF25-7 and BSDF48-3, exceeding 5 × 10^8^ colony-forming units/cm^2^ on stainless steel coupons. In contrast, the highest biofilm biomass accumulated was BSDF2-3 and BSDF25-7, indicating differences in cell proliferation and biofilm matrix structure. CryoSEM imaging revealed diverse biofilm structures, such as silo-like aggregates and patchy surface colonization, indicating strain-specific extracellular matrix assembly strategies. Flow-through biofilm bioreactor assays further identified BSDF2-3 and BSDF5-1 as predominant biofilm formers with the highest CFU/cm^2^ present at 4 × 10^8^ and 1 × 10^9^, respectively, while BSDF2-3 accumulated twice the biofilm biomass as BSDF5-1. *Leuconostoc* strains BSDF25-7 and BSDF48-3 produced high levels of dextran and EPS, while BSDF2-3 consistently formed dense, shear-resistant biofilms despite slow growth and low EPS levels, suggesting the possibility of alternative matrix composition or structural adaptations. Individual *Leuconostoc* strains adapt uniquely, adding to the functional diversity of biofilms that impact formation, matrix complexity, and resistance to environmental stressors. This study furthers our understanding of EPS and growth phenotypes involved in biofilm formation while providing a working model, enabling the development of future antimicrobial mitigation strategies.

## 1 Introduction

Sugar beet (*Beta vulgaris ssp. vulgaris*) is a vital crop, contributing significantly to global sugar production. The United States is among the top global producers contributing considerably to the sugar economy, with a projected 5.389 million short tons raw value (STRV) for the 2024/2025 campaign (Abadam, 2025). During post-harvest management of sugar beet processing, microbial contamination originating from sources, such as infected roots and associated soil, storage piles, juice (Bill et al., 2024; Bill et al., 2025), and factory biofilms (Qi et al., 2023; Qi et al., 2025), leads to sucrose losses and operational challenges (Soliman, 2007; Fugate and Campbell, 2009; Eggleston et al., 2011; Ernst et al., 2024). Microorganisms introduced from infected sugar beet roots, soil, and storage piles are carried into the processing streams, leading to sucrose deterioration and formation of EPS that interfere with clarification, clogging of filters, increased viscosity, and inhibition of sugar crystallization (Abdel-Rahman et al., 2008; Borji et al., 2019; Evageliou, 2020; Abdel-Rahman et al., 2023).

*Leuconostoc* spp., along with several other bacteria and fungi, such as *Lactobacillus*, *Bacillus, Clostridium*, *Candida*, *Fusarium*, and *Penicillium,* are frequently encountered in sugar beet processing environments (Kusstatscher et al., 2019; Kusstatscher et al., 2021; Bill et al., 2025; Qi et al., 2025). These microorganisms, especially lactic acid bacteria (LAB), metabolize sucrose into organic acids (Mendonça et al., 2022) and EPS, leading to pH shifts, reduced sucrose purity, and the accumulation of viscous byproducts in juice processing lines. EPS, such as dextran, levan, and heteropolysaccharides, can block filters, increase viscosity, and disrupt crystallization during downstream refining (Hector et al., 2016; Borji et al., 2019; Dolgopolova et al., 2021; Qi et al., 2023; Bruni et al., 2024). The resulting processing inefficiencies not only cause sugar losses but also necessitate costly interventions, such as frequent maintenance, enzyme treatments, and antimicrobial usage.

Although dextranase is routinely added during processing to target dextran, primarily produced by *Leuconostoc* and *Lactobacillus,* persistent processing issues suggest that other types of EPS may also be involved (Eggleston et al., 2009; Nel et al., 2019; Misra et al., 2022). Moreover, EPS, in addition to proteins, may facilitate adherence to factory surfaces during biofilm formation, further complicating cleaning and sanitation efforts. While there are several reports on EPS produced by *Leuconostoc* (Leathers and Côté, 2008; Côté and Leathers, 2009; Yan et al., 2016; Zikmanis et al., 2020), there are relatively few studies on biofilm formation in sugar crop factories or with factory-derived isolates, although some reports suggest that biofilms in sugarcane and sugar beet factories are polymicrobial in nature and encased in polysaccharides (Leathers and Côté, 2008; Qi et al., 2023; Qi et al., 2025). These studies also suggest the association of lactic acid bacteria and yeast in many biofilm samples (Qi et al., 2023; Qi et al., 2025).

The extracellular matrix of biofilms encases and shields the microbial community from external stresses such as heat and chemical treatments, making them more resistant to biocides and promoting persistent contamination (Liu et al., 2023; Liu et al., 2024). The combination of EPS-mediated viscosity issues and biofilm-related biofouling presents a multifaceted obstacle to efficient sucrose recovery in factories. Therefore, elucidating the phenotypic diversity in growth rate, EPS production, and biofilm biomass accumulation among *Leuconostoc* strains is central to understanding mechanisms of biofilm formation in sugar crop factories and developing effective control measures. This study sought to highlight the variation in biofilm production among nine *Leuconostoc* strains obtained from juice and biofilm niches within sugar beet factories (Qi et al., 2025). While earlier studies have modeled biofilm formation under high fluid shear with one strain at a time (Leathers and Côté, 2008; Côté and Leathers, 2009), this study utilized a biofilm bioreactor with low fluid shear and six growth chambers, enabling comparison of multiple strains and production of greater biofilm biomass for the study. Assessment of biofilm-related parameters indicates that varying phenotypic traits among *Leuconostoc* strains impact biofilm formation.

## 2 Materials and methods

### 2.1 Bacterial strains and growth media

*Leuconostoc* strains were isolated and identified from sugar beet factory juice and biofilm samples as described (Qi et al., 2025). The whole genome sequencing of these strains was previously reported (Joshi and Bruni, 2025). All strains were grown as precultures in MRS medium (De Man et al., 1960) at 28 °C, 250 rpm, unless specified otherwise. The strains used in this study are shown in Table 1.

**Table 1:** *Leuconostoc* isolates used in this study were previously described (Joshi and Bruni, 2025; Qi et al., 2025).

Strains	Source (sugar beet factory)	Identification
BSDF2-3	Biofilm	*Leuconostoc suionicum*
BSDF2-6	Biofilm	*Leuconostoc suionicum*
BSDF62-9	Juice	*Leuconostoc mesenteroides*
BSDF47-1	Juice	*Leuconostoc suionicum*
BSDF48-3	Juice	*Leuconostoc suionicum*
BSDF14-9	Biofilm	*Leuconostoc suionicum*
BSDF52-11	Juice	*Leuconostoc suionicum*
BSDF5-1	Biofilm	*Leuconostoc citreum*
BSDF25-7	Biofilm	*Leuconostoc suionicum*

### 2.2 Batch adherence phase

Biofilms were grown in a Drip-flow six-chamber biofilm reactor (Biosurface Technologies, Bozeman, MT, USA). Each strain was cultured overnight in 50 mL of MRS medium (De Man et al., 1960) in a 250-mL flask at 28 °C, with shaking at 250 rpm. Bacterial cells were normalized to approximately 5 × 10^8^ colony-forming units (CFU) per mL, expressed as CFU/mL in Tryptone Sucrose Yeast (TSY) medium, 120 g/L sucrose, adapted from Haynes et al. (1955), and inoculum cell counts were confirmed by plating for CFU/mL (see next section). 1 mL of normalized bacterial culture was inoculated into 15 mL of TSY medium in each growth chamber. The growth chambers were clamped at the effluent port to retain the growth medium during the batch adherence phase. Then 0.22-μm filter vents were attached to allow for aerobic growth. The inoculated biofilm reactor was incubated at 28 °C for 48 h during the batch phase to facilitate adherence to stainless steel coupons. Samples were then taken for quantification of microbial CFU/cm^2^ recovered from stainless steel coupons and measurement of biofilm dextran (see below).

### 2.3 Flow-through bioreactor analysis

For flow-through bioreactor experiments, *Leuconostoc* spp. were processed similarly to the adherence phase experiment for 48 h. After 48 h, continuous flow was initiated at room temperature with a flow rate of 0.9 mL/min using TSY medium (120 g/L sucrose). The bioreactor was attached to a shaker platform with a speed setting of 8 rpm with front-to-back shaking (Fisherbrand, Waltham, MA, USA). The bioreactor was secured to the shaker platform with bungee cords. The continuous flow-through phase was maintained for 72 h. Then, samples were collected for the quantification of microbial CFU/cm^2^ and biofilm biomass.

### 2.4 Quantification of microbial colonies

Samples for both adherence and continuous flow biofilm material were removed from the topside of stainless-steel coupons using a sterile cell scraper into 30 mL sterile ultrapure water. Biofilm biomass was then homogenized using an Ika T10 homogenizer (Ika Works, NC, USA) at 20,000 rpm for 30 s. Biofilm homogenates were serially diluted in sterile ultrapure water and plated onto MRS agar with an Eddy Jet 2 W Automatic Spiral Plater in triplicate in linear 100 mode (Neutec Group, Inc., NY, USA). Plates were incubated at 28 °C for 1–2 days until colonies appeared. Colonies were counted with the SphereFlash Automatic Colony Counter (Neutec Group, Inc., NY, USA) using the SphereFlash Colonies PRO software version 1.0.0.16. to measure CFU/mL.

CFU/mL from triplicate plates were averaged and divided by the area of the stainless-steel coupon, 18.75 cm^2^, resulting in CFU/cm^2^ metrics for each growth chamber. Each strain was run in side-by-side duplicate growth chambers. The resulting CFU/cm^2^ from duplicate growth chambers was averaged for each strain. *L. suionicum* BSDF2-3 was used as a positive control for each experiment, allowing for additional experimental strains to be run in duplicate during each experiment since the bioreactor has a total of six side-by-side growth chambers. Each experiment was run at least twice.

### 2.5 Biofilm biomass

For both adherence and continuous flow experiments, biofilm biomass was precipitated with three volumes of absolute ethanol at 4 °C overnight. Samples were then centrifuged at 10,000×*g* at 4 °C for 30 min to pellet the biofilm biomass. The resulting pellet was lyophilized, weighed, and stored in a desiccator under vacuum.

### 2.6 Scanning electron microscopy (SEM) imaging

To examine the adherence phase biofilms formed by each isolate, a thin layer of biofilm sample was mounted on aluminum SEM stubs using a cryo-embedding compound (Pelco, Ted Pella, CA, USA). The prepared stubs were placed on a temperature-controlled stage (Coolstage, Deben, UK), which was maintained at −20 °C throughout imaging to minimize dehydration and preserve surface integrity. Then, the samples were analyzed with a Phenom G6 ProX SEM (Nanoscience Instruments, Phoenix, AZ, USA) operated under low-vacuum conditions to accommodate the partially hydrated state of the biofilms. SEM images were acquired at accelerating voltages of 5 kV and 10 kV, with magnifications of 2,000× and 5,000×, respectively.

### 2.7 Dextran assay

Biofilm samples harvested from adherence experiments were homogenized in 30 mL of sterile ultrapure water, serially diluted, and analyzed for dextran content using a dextran enzyme-linked immunosorbent assay (ELISA) kit (Beacon Analytical Systems, Saco, ME, USA). The calibration curve and sample data were processed according to the manufacturer’s recommendations. Data were analyzed using 4-parameter logistic (4PL) regression, and dilution factors were taken into consideration for each isolate.

### 2.8 Viscosity and exopolysaccharides measurements

*Leuconostoc* strains were grown, and the EPS was prepared as previously described (Bruni et al., 2024). Briefly, each strain was grown overnight as a preculture in MRS medium (De Man et al., 1960) and then inoculated into flask cultures at a starting OD of 0.05 in 50 mL TSY medium. Cultures were grown for 24 h at 28 °C with 250 rpm shaking. These flask cultures were used for both EPS and viscosity measurements.

After the 24-h incubation, OD_600_ was measured with an Eppendorf Bio-Photometer (Hamburg, Germany), and flask culture viscosity was measured with a Brookfield model DV-11 + viscometer. Viscosity measurements were conducted using approximately 20 mL sample volume withdrawn from the flask culture, without dilution, using a UL spindle and small sample adapter at room temperature (Qi et al., 2025). Complete results from the viscosity analysis are given in the Supplementary material S1. One viscosity measurement was performed on three replicate culture flasks for each strain. For each sample, where possible, viscosity was measured at three different rotational speed instrument settings in the range of 5 to 50 RPM. Three samples, BSDF (5–1, 2–6, and 2–3), were substantially more viscous than other samples and required measurement at a rotational speed of 0.5 RPM (minimum instrument setting). For all other samples BSDF (48–3, 14–9, 47–1, 25–7, 62–9, 52–11), the recorded viscosity was taken as the measured viscosity at the lowest instrument speed setting, where the measured torque output exceeded a value of 10%; instrument manufacturer recommendation is to only use viscosity readings with associated torque values between 10 and 100%. Although shear thinning phenomena have been observed for some samples of this nature in a previous study (Qi et al., 2025), we assume Newtonian fluid behavior (Evageliou, 2020) here as a simplifying assumption in order to enable practical comparison between results and subsequent correlational analyses.

For EPS measurement, samples were centrifuged to remove bacterial cells, and supernatants containing soluble EPS were precipitated with three volumes of ethanol without DNase or proteinase treatment at 4 °C overnight. EPS was pelleted by centrifugation at 10,000 *g* for 30 min, and then lyophilized, weighed, and stored in a desiccator under vacuum (Bruni et al., 2024).

### 2.9 Doubling time determination

Precultures of each isolate were grown in MRS medium (De Man et al., 1960) to minimize EPS production and allow for pipetting non-viscous precultures. Optical densities (OD_600_, hereafter OD) of precultures were measured in an Eppendorf Bio-Photometer (Hamburg, Germany) and normalized to 0.2 in TSY medium (50 g/L sucrose) (Haynes et al., 1955). Normalized precultures were pipetted into a 96-well plate in triplicate. A block design was employed to arrange the microbial isolates within the plates, and each block included triplicate blank wells without inoculum to serve as a reference. Plates were incubated at 28 °C with shaking at 250 rpm for 24 h in a TECAN plate reader (TECAN, Männedorf, Switzerland), which recorded OD values every 10 min using Magellan Standard software, version 7.5.

The Richards growth model was fitted to modified curves of the samples (inoculated wells). First, OD values from blank wells were subtracted from those of inoculated wells to generate adjusted OD values. To ensure accurate modeling of the exponential growth phase, data points occurring after the first instance of 99% of the maximum OD value for each curve were excluded, preventing the Richards growth model from overfitting the stationary phase. All model fitting was performed in R version 4.4.1 (Team R.C., 2016). The Richards growth model was fitted to these modified growth curves using the Richards growth function from the R package growth rates version 0.8.4 (Petzoldt, 2022). Visual inspection of the fits was conducted using the ggplot2 R package (Gómez-Rubio, 2017).

The maximum growth rate (mumax) was extracted from the fitted Richards growth equations (Richards, 1959). This value was then used to calculate the doubling time (*t*) using the exponential growth equation, *N*(*t*) = *N*(0)e^(*rt*)^, where *N*(*t*) is the final abundance, *N*(0) is the initial abundance, and ‘e’ is Euler’s number. The doubling time for each isolate was determined by setting *N*(*t*) = 2, *N*(0) = 1, and *r* = mumax, and solving for *t*. The doubling times derived for triplicate samples of each isolate were subsequently averaged.

### 2.10 Statistical analysis

Statistical analysis was conducted using GraphPad Prism Software (version 10.4.2, GraphPad Software, United States). The data represent at least two independent trials and are shown as the mean ± standard error. To compare multiple groups, a one-way ANOVA test was used, followed by a Tukey *post hoc* test. Significant differences were considered at *p*-values less than 0.05. Similarly, correlation analysis was performed using the Pearson R correlation test.

## 3 Results

### 3.1 Variation in adhesion, proliferation, and biomass accumulation among *Leuconostoc* isolates

**Figure 1:**
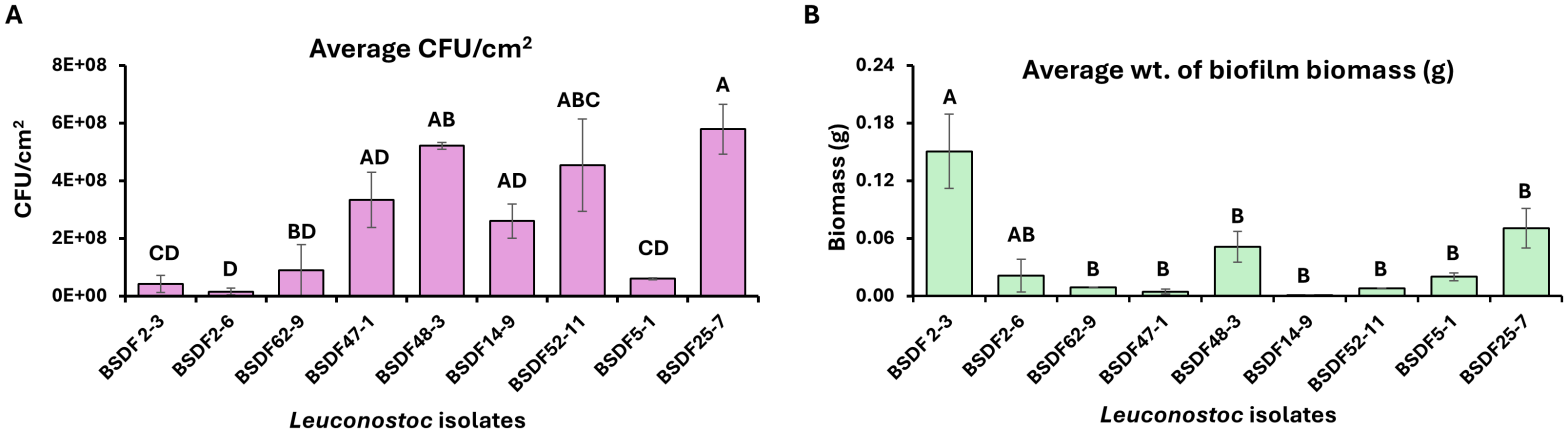
Biofilm formation by *Leuconostoc* isolates in a 48 h batch phase to measure adhesion and proliferation on stainless steel coupons was measured as **(A)** the average CFU/cm^2^ and **(B)** average dry biomass (g) of biofilm. Values are based on at least two independent experiments with two coupons per experiment. Significant differences between isolates (*p* < 0.05) were determined by ANOVA followed by Tukey *post-hoc* analysis as indicated by distinct letters.

*Leuconostoc* strains isolated from juice and biofilm samples in sugar beet factories were examined for the ability to adhere, proliferate on abiotic surfaces, and form biofilms. This was assessed through quantification of viable cell counts and biofilm biomass after 48 h of the batch phase (Figures 1A,B and Supplementary material S2). All nine strains exhibited the capacity to adhere and proliferate on the abiotic surface, but with significant variation in CFU density and biomass accumulation across isolates. In Figure 1A, average CFU/cm^2^ measurements indicated that strains *L. suionicum* BSDF25-7 and BSDF48-3 exhibited significantly higher adhesion and proliferation, each exceeding 5 × 10^8^ CFU/cm^2^, while strains such as *L. suionicum* BSDF2-3, BSDF2-6, *L. mesenteroides* BSDF62-9, and *L. citreum* BSDF5-1 showed markedly lower CFU levels at 10^7^ CFU/cm^2^. However, biofilm biomass quantification (Figure 1B) revealed that BSDF2-3 formed significantly denser biofilm matrices compared to other strains. Notably, strains such as *L. suionicum* BSDF25-7 and BSDF48-3 displayed comparatively low biomass, indicating potential differences in extracellular matrix production relative to cell density. Statistical analysis using one-way ANOVA followed by Tukey’s post hoc test confirmed significant differences among strains in both CFU and biomass measures. These findings highlight substantial strain-specific heterogeneity in biofilm-forming phenotypes, suggesting possible differences in cell surface properties, EPS production, or regulatory mechanisms underlying biofilm development in *Leuconostoc* isolates.

### 3.2 Biofilm formation in a flow-through bioreactor reveals strain-dependent variation

**Figure 2:**
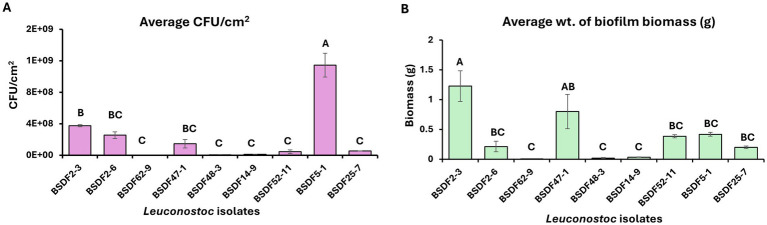
Biofilm formation by *Leuconostoc* isolates in a 72 h continuous flow phase to measure proliferation and biomass accumulation on stainless steel coupons, measured as **(A)** the average CFU/cm^2^ and **(B)** average dry biomass (g) of biofilm. Values are based on at least two independent experiments, with two coupons per experiment. Significant differences between isolates (*p* < 0.05) were determined by ANOVA followed by Tukey *post-hoc* analysis as indicated by distinct letters.

The ability of *Leuconostoc* strains to proliferate and form biofilms in a continuous flow bioreactor was evaluated in a flow-through bioreactor system for 72 h. Quantification of surface-associated viable cells (CFU/cm^2^) and total biofilm biomass showed clear strain-level differences in biofilm formation (Figures 2A,B).

*L. citreum* BSDF5-1 had the highest 1.14 × 10^9^ CFU/cm^2^ in contrast to *L. mesenteroides* BSDF62-9, which was the lowest biofilm-forming strain with 1.33 × 10^6^ CFU/cm^2^ (*p* value <0.0001), as shown in Figure 2A. Among *L. suionicum*, BSDF2-3 showed the highest count of 3.73 × 10^8^ CFU/cm^2^, followed by BSDF2-6 with 2.55 × 10^8^ CFU/cm^2^. These data suggest bacterial proliferation leading to increased CFU counts in 72 h. Biofilm biomass accumulation during the continuous flow-through phase showed trends similar to the batch phase biofilm biomass phenotypes. The highest average biofilm biomass was produced by *L. suionicum* BSDF2-3 compared to all other strains. However, *L. suionicum* BSDF47-1 maintained low CFU/cm^2^ levels but produced moderate biomass. The minimal biofilm biomass produced by *L. mesenteroides* BSDF62-9 also corresponded with the low CFU/cm^2^ values (Figure 2B).

### 3.3 Topological variation in *Leuconostoc* biofilm shows aggregation and pillar-like architectures

**Figure 3:**
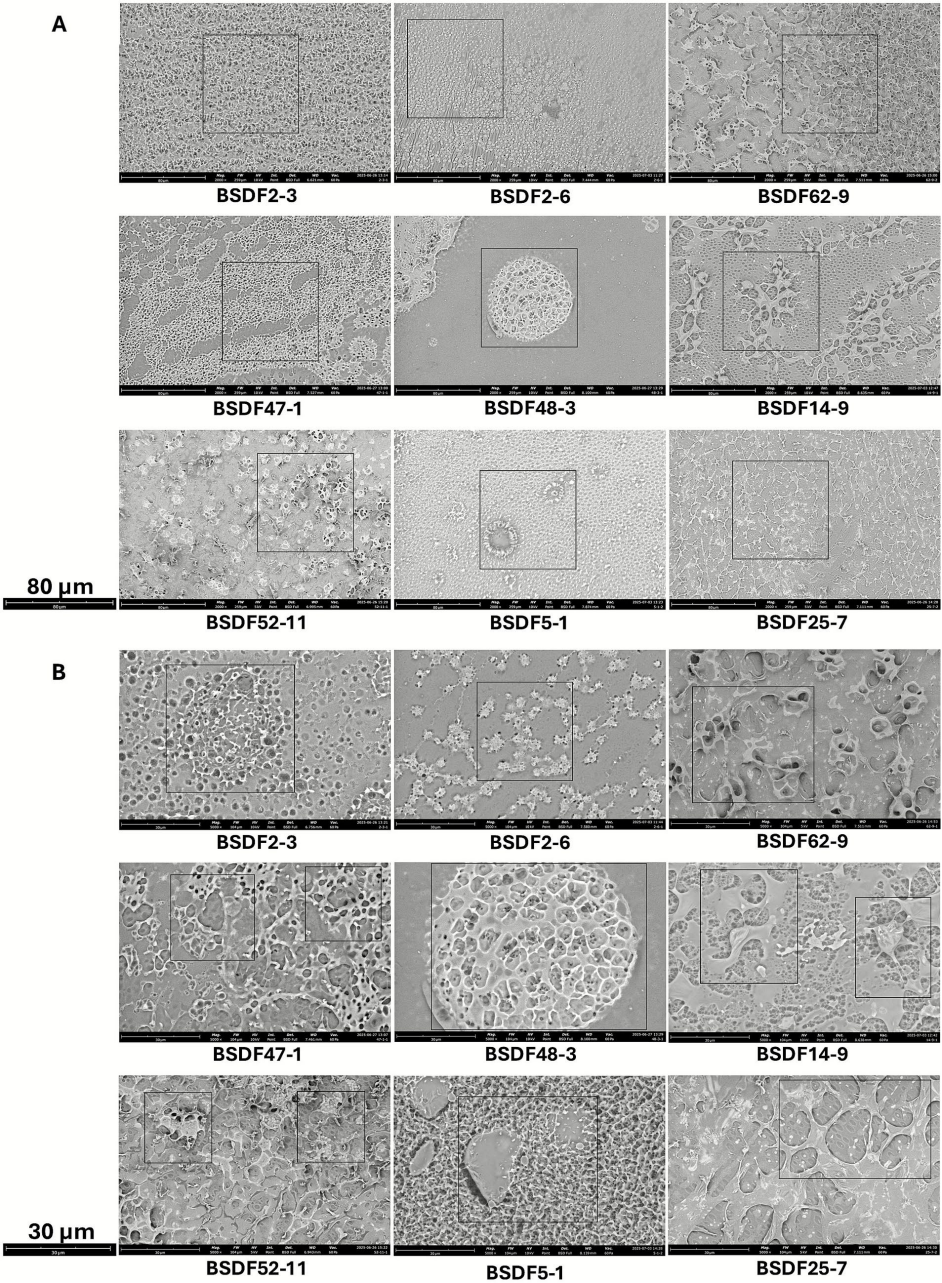
Scanning electron microscopy images of a 48-h biofilm from the adhesion batch phase experiment. Images were taken on a Phenom G6 ProX SEM with **(A)** 2,000× and **(B)** 5,000× magnifications.

SEM images revealed significant topological diversity in 48-h cultivated biofilm structures (Figures 3A,B and Supplementary material S3). At 2,000× magnification (Figure 3A), *L. suionicum* strains BSDF2-3, BSDF2-6, and BSDF47-1 formed dense, mat-like biofilms with relatively uniform surface coverage. In contrast, *L. suionicum* BSDF48-3 and *L. citreum* BSDF5-1 formed distinct, silo- or dome-like protrusions emerging from a sparsely colonized background. BSDF62-9 showed weak biofilm formation, presenting a loose, watery structure. Notably, BSDF48-3 exhibited a large, centrally aggregated pillar structure, suggesting vertical stratification and localized microcolony development. These elevated morphologies resemble the “mushroom” or “silo” structures commonly seen in mature biofilms of *Pseudomonas* and *Staphylococcus*, which are linked to nutrient gradients and protective EPS layers (Coraça-Huber et al., 2020; Wang et al., 2023).

At higher magnification (5,000×, Figure 3B), finer structural details became evident. *Leuconostoc* strains, such as BSDF62-9 and BSDF14-9, showed coarse, corrugated, net-like surfaces with loosely aggregated cells. In contrast, strains BSDF2-6 and BSDF52-11 exhibited sparse cell clusters interspersed with voids, indicating limited matrix formation and weaker cohesion. The strain BSDF48-3 showed a compact, elevated central mass, composed of tightly packed cells arranged radially, possibly indicating increased localized EPS deposition. Notably, strains *L. citreum* BSDF5-1 and *L. suionicum* BSDF2-3 demonstrated a mucinous, spiky monolayer-like conformation with vertical growth, suggesting a biofilm adapted for surface adhesion rather than volume expansion.

### 3.4 Strain-specific variation in the growth rate of *Leuconostoc* isolates

**Figure 4:**
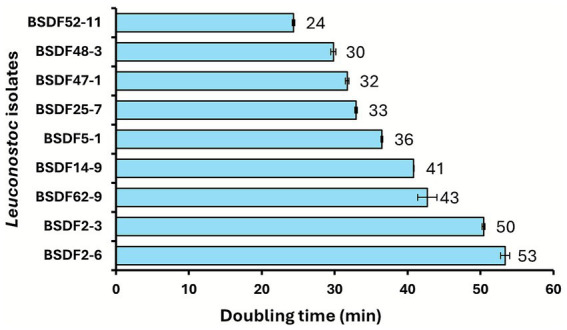
Average doubling time of *Leuconostoc* sp. isolates arranged from fastest (top) to slowest (bottom). Doubling times were calculated from growth curves inoculated at 0.2 OD_600_, using growth rate *r* from fitted Richards growth models. Sample size *n* = 3. Error bars are the sample standard error.

Next, in order to determine whether bacterial proliferation rate may impact biofilm formation, the doubling time of each *Leuconostoc* strain was determined under standard growth conditions to establish if biofilm formation phenotypes may be attributable to inherent growth rates (Figure 4). Substantial variation in growth rates was observed across all *Leuconostoc* strains, with doubling times ranging from approximately 24–53 min. *L. suionicum* strains BSDF52-11 and BSDF48-3 exhibited the fastest growth rates, with doubling times under 30 min, while *L. suionicum* strains BSDF2-3 and BSDF2-6 showed slower growth, with doubling times exceeding 50 min.

### 3.5 Strain-dependent production of dextran in batch phase biofilms

**Figure 5:**
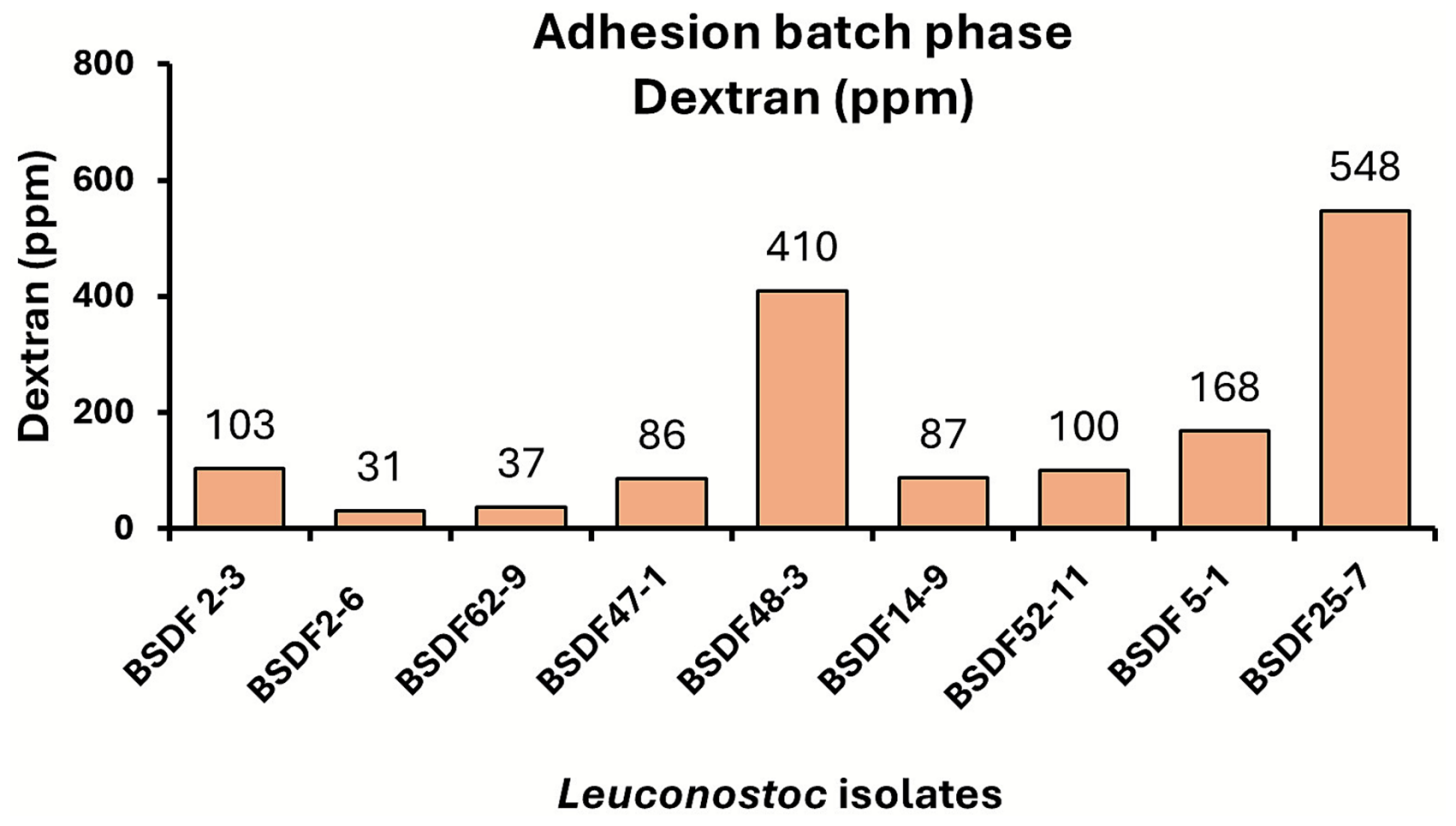
Concentration of dextran (ppm) from the adhesion batch phase biofilm. Dextran ELISA was performed with standard calibrations, and samples were run in triplicate. Dextran ELISA kit 4-parameter logistic regression curve showed *R*^2^ = 0.99.

To evaluate the EPS production capacity among *Leuconostoc* strains, dextran levels were quantified from batch-phase biofilm samples using a dextran ELISA kit and analyzed using a 4-parameter logistic regression model (*R*^2^ = 0.99; Figure 5). Substantial differences in dextran concentrations were observed among the nine *Leuconostoc* strains, with values ranging from approximately 31 to over 548 ppm. In particular, *L. suionicum* strains BSDF25-7 and BSDF48-3 exhibited the highest dextran production, consistent with high CFU/cm^2^ densities and biomass accumulation observed in the batch-phase experiments. In contrast, strains such as *L. suionicum* BSDF2-6 and *L. mesenteroides* BSDF62-9 produced significantly lower levels of dextran. These results highlight extensive heterogeneity in dextran synthesis among *Leuconostoc* strains and underscore the potential contribution of dextran production to the robustness of biofilms.

### 3.6 Quantification of EPS and viscosity reveals variability in the extracellular matrix yield

**Figure 6:**
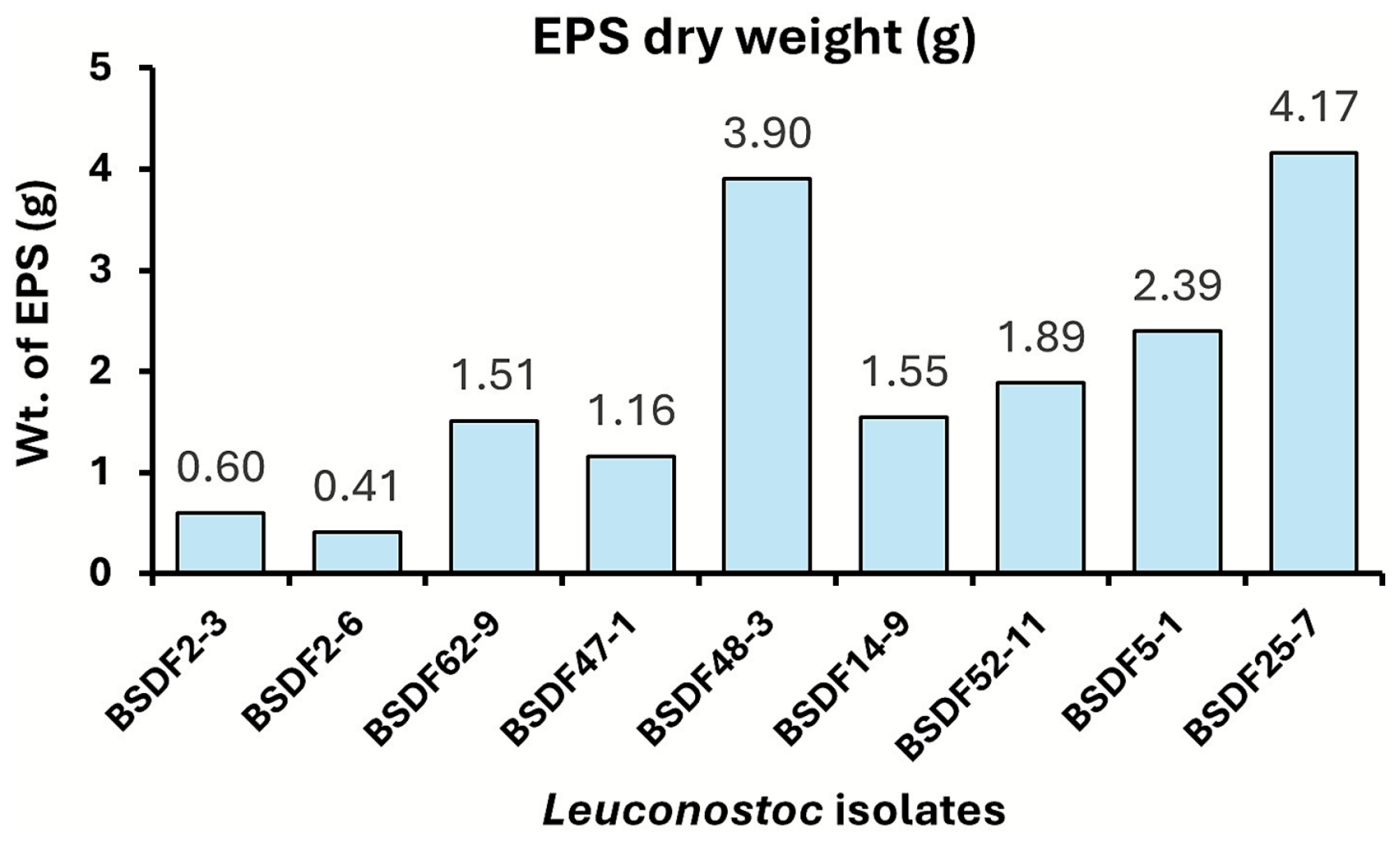
Dry weight (g) of EPS from *Leuconostoc* isolates. EPS was extracted from 24 h flask cultures. Sample flasks were run in duplicates and pooled.

EPS and viscosity were measured from 24-h *Leuconostoc* flask cultures to gain a comprehensive understanding of *Leuconostoc* strain EPS production capacity under planktonic conditions (Figure 6 and Table 2). Substantial variation was observed in EPS yield dry weight extracted from the flask cultures among the strains, with values ranging from less than 0.5 g to over 4 g per culture. Strains BSDF25-7 and BSDF48-3 produced the highest amounts of EPS, indicating strong extracellular matrix production under planktonic batch growth conditions. In contrast, strains BSDF2-3 and BSDF2-6 exhibited markedly lower EPS levels, which may be attributed to variations in EPS solubility when preparing EPS samples from cultures. Notably, others have reported that some *Leuconostoc* isolates produce insoluble EPS fractions that remain cell-associated (Padmanabhan and Kim, 2002; Côté and Leathers, 2009). This possibility and the impact of EPS structure on solubility warrant further investigation. The differences in EPS yield broadly align with trends observed in the dextran ELISA results and biofilm biomass accumulation, supporting the notion that EPS production is a key factor in strain-specific biofilm phenotypes.

**Table 2:** Average viscosity and measured OD_600_ of *Leuconostoc* 24-h flask cultures.

*Leuconostoc* isolates	Avg. viscosity cPs	Avg. viscosity (log_10_) cPs	Avg. OD_600_
BSDF2-3	1,095 ± 35	3.04 ± 0.01	7.10 ± 0.42
BSDF2-6	1,200 ± 10	3.08 ± 0.01	6.25 ± 0.32
BSDF62-9	5.63 ± 0.34	0.75 ± 0.02	2.03 ± 0.01
BSDF47-1	3.03 ± 0.04	0.48 ± 0.01	2.47 ± 0.01
BSDF48-3	10.8 ± 0.3	1.03 ± 0.01	2.79 ± 0.05
BSDF14-9	3.08 ± 0.04	0.49 ± 0.01	2.42 ± 0.03
BSDF52-11	3.42 ± 0.02	0.53 ± 0.01	2.83 ± 0.03
BSDF5-1	639 ± 1	2.79 ± 0.07	5.61 ± 0.23
BSDF25-7	10.8 ± 0.2	1.03 ± 0.01	2.69 ± 0.01

Data are presented as mean ± standard error.

Furthermore, viscosity measurements demonstrated that strains exhibited varying levels of viscosity in the respective flask culture broths (Table 2). Since measured viscosities span orders of magnitude, values are reported both as—is and as log_10_—transformed. Due to the wide variation in culture viscosity, culture samples were read at different rpm in order to obtain viscosity measurements with a torque greater than 10. *L. suionicum* BSDF2-3 and BSDF2-6 strains produced the highest average viscosity, measured at 3.04 ± 0.01 log_10_ and 3.08 ± 0.01 log_10_ cP, respectively. *L. citreum* BSDF5-1 exhibited the next highest viscosity of 2.79 ± 0.07 log_10_ cP (Table 2). The remaining *Leuconostoc* isolates had lower viscosities ranging between 0.48 and 1 cP. Taken together, the results indicate that *Leuconostoc* strains possess diverse capabilities in modifying the viscosity of the growth environment through the production of various EPS.

### 3.7 Dry-weight EPS is positively correlated with dextran concentration

**Figure 7:**
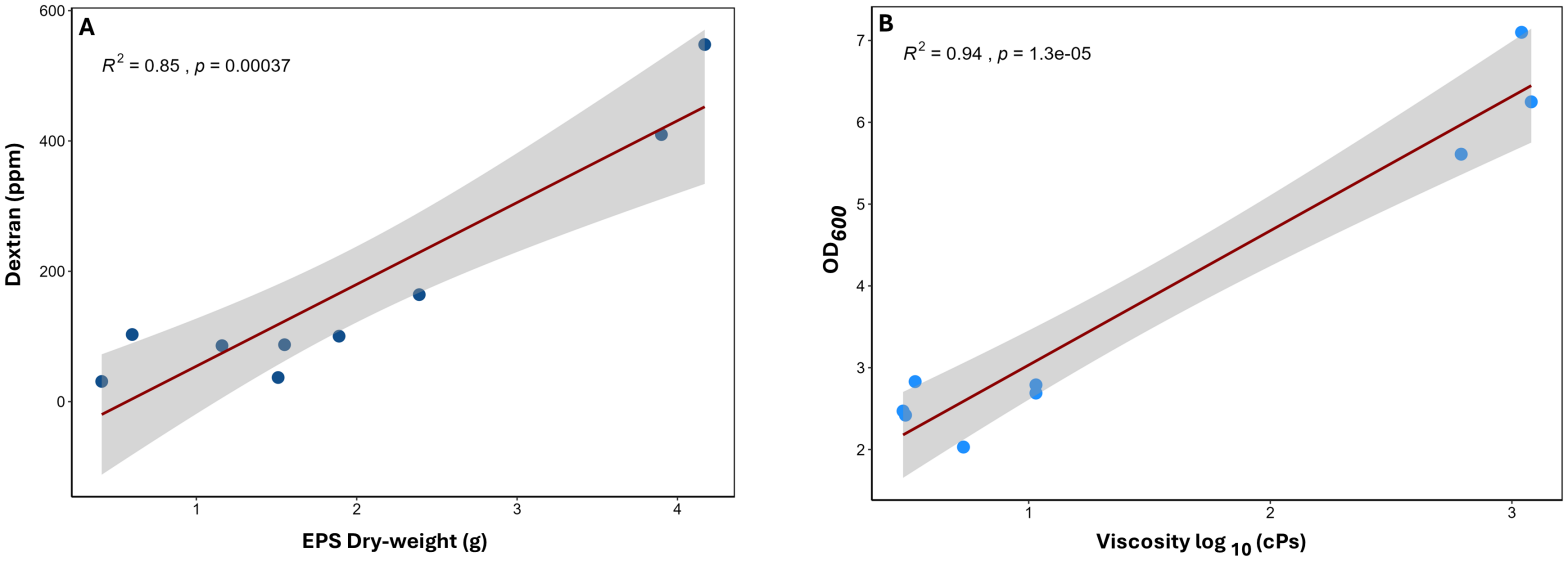
Scatter plot showing the correlation between **(A)** EPS and dextran and **(B)** viscosity and OD_600_ by Pearson correlation from 24-h *Leuconostoc* flask cultures.

Finally, the functional contribution of the EPS production capacity of the respective *Leuconostoc* strains was assessed. Pearson correlation was performed between the dextran levels measured in batch phase biofilms and the extracted EPS dry weight of flask cultures (Figure 7A) since the biofilm biomass from the adherence phase was very low for some strains. A significant positive correlation (*R*^2^ = 0.85, *p* = 0.00037) was observed between the EPS weight and the dextran concentration, indicating that strains with greater EPS yields also produced higher quantities of dextran (Figure 7A).

Next, Pearson correlation was performed for the viscosity of the 24-h flask cultures and OD_600_ (Figure 7B). Similarly, a significant positive correlation was found (*R*^2^ = 0.94, *p* = 0.000013), suggesting that OD_600_ contributes to the rheological properties of the culture medium (Figure 7B). Interestingly, the observed viscosities showed poor correlation with EPS yield (Figure 6), suggesting additional variables such as OD_600_, EPS structure, and solubility may also impact viscosity. These findings highlight the central role of strain-specific EPS biosynthesis in modulating both biochemical (dextran content) and biophysical (viscosity) characteristics of *Leuconostoc* cultures.

## 4 Discussion

*Leuconostoc* species are key microbial contaminants of post-harvest sugar beet processing streams, often originating from rotting sugar beet roots and subsequently contaminating the factory (Bill et al., 2024; Qi et al., 2025). These microbes pose significant issues for raw sugar production, such as sucrose losses and operational disruptions during processing (Qi et al., 2023; Bill et al., 2025; Qi et al., 2025). An examination of the phenotypic variability in growth, EPS, and biofilm-forming capacity of these *Leuconostoc* strains originating from the sugar beet factory environment is presented in this study. Notably, *Leuconostoc* strains BSDF2-3, BSDF2-6, BSDF14-9, and BSDF5-1 were originally isolated from sugar beet factory biofilm samples, while *Leuconostoc* strains BSDF48-3, BSDF62-9, BSDF52-11, and BSDF47-1 were isolated from sugar beet factory juice samples (Qi et al., 2025). Additionally, the genome sequences of the nine *Leuconostoc* isolates are reported (Joshi and Bruni, 2025).

Our study found significant heterogeneity in biofilm-forming capabilities, EPS production, and growth kinetics among *Leuconostoc* isolates from industrial sugar beet factories. Under both static and dynamic culture conditions, the isolates varied greatly in adhesion, CFU density, and total biomass during both the batch phase and the continuous flow through phase. Notably, strains BSDF25-7 and BSDF48-3 showed strong planktonic growth and surface colonization under batch conditions. At the same time, BSDF2-3 formed dense biofilm matrices despite lower CFU counts during the batch phase, suggesting that biomass accumulation is not solely reliant on cell proliferation (Figure 1). This decoupling likely reflects different regulatory strategies controlling the biofilm matrix and cellular division, as seen in other bacterial systems (Flemming and Wingender, 2010; Flemming et al., 2016; Karygianni et al., 2020). In flow-through assays simulating industrial shear stress, BSDF5-1 and BSDF2-3 maintained high biomass and CFU, indicating biofilm resilience under hydrodynamic stress (Figure 2).

Similar phenomena have been observed in *Pseudomonas* spp., where shear exposure induces biofilms with higher carbohydrate content and better structural stability, increasing resistance to detachment (Dingemans et al., 2016; Palalay et al., 2023). BSDF2-3 proved to be a strong biofilm former in both models, despite slower growth and lower EPS yield by mass. Conversely, BSDF25-7, despite its strong adhesion during the batch phase, showed poor adherence under fluid shear, highlighting the importance of testing biofilms in conditions that resemble those in industrial pipelines for evaluating persistence. Substantial variability in the composition and structure of EPS may be partially attributable to various factors, such as additional extracellular components, for example, DNA and protein, as well as other microbes in the community, the ambient shear forces, nutrient and substrate availability, and the characteristics of the host environment (Lebeaux et al., 2014; Flemming et al., 2016).

Scanning electron microscopy of biofilm samples revealed considerable phenotypic variability in the three-dimensional architecture (Figure 3). The diverse topographical features of *Leuconostoc* biofilms pose significant challenges during sugar beet processing by decreasing sanitation efficiency. Filter blockages in processing streams are also a significant operational challenge (Qi et al., 2023; Qi et al., 2025). Strains such as BSDF48-3 and BSDF5-1 exhibited distinct, silo-like structures, while strain BSDF62-9 displayed irregular, loosely aggregated matrices. Notably, the elevated, EPS-rich structures formed by some *Leuconostoc* strains like BSDF48-3 and BSDF5-1 resemble mature biofilms seen in other LAB and pathogens, promoting long-term persistence and antimicrobial tolerance (Costerton et al., 1995). Conversely, flattened, fragmented biofilms likely indicate weaker matrix formation or impaired adhesion, reducing survival under shear stress and posing less threat in dynamic systems (Simões et al., 2010; Paul et al., 2012; Simões and Simões, 2023).

This structural diversity likely reflects differences in EPS structural composition as well as any accompanying extracellular DNA and proteins that may affect adhesion dynamics. These features may affect strain-specific biofilm resilience, nutrient diffusion, and persistence in industrial settings.

Growth rate analysis provided further insight into the phenotypic divergence among strains. Fast-growing strains, such as BSDF48-3 and BSDF52-11, exhibited shorter doubling times and higher CFU levels during the batch phase, whereas slow-growing strains, like BSDF2-3 and BSDF2-6, showed increased biofilm biomass during the continuous flow phase and increased viscosity during planktonic culture, indicative of a biofilm-specialized phenotype (Figure 4 and Table 2). This tradeoff between rapid proliferation and matrix investment supports patterns observed in LAB and other gram-positive bacteria, where biofilm specialization involves resource allocation toward structural polymer synthesis rather than cell division (Niederdorfer et al., 2017; Reichhardt et al., 2025). In contrast, BSDF2-3 forms higher-biomass biofilms during the continuous flow phase with modest EPS recovery, indicating a more compact, cross-linked matrix likely enriched in insoluble or branched polysaccharides (Leathers and Côté, 2008; Côté and Leathers, 2009).

Additionally, the divergence in observed strain-specific biofilm phenotypes may reflect ecological adaptation to niches within the sugar-processing environment, where selection pressures such as flow, surface contact, and nutrient flux drive EPS diversity (Hector et al., 2016; Henriksen et al., 2022). Notably, strains with faster doubling times also tended to form more viable cells during the adherence batch phase when nutrients are limited, while slower-growing strains, such as *L. suionicum* BSDF2-3 and *L. citreum* BSDF5-1, tended to be robust biofilm producers during the continuous-flow phase with unlimited sucrose. These findings indicate that growth kinetics may partially underpin the observed strain-specific variation in biofilm phenotypes.

Quantitative analyses revealed considerable variation in total EPS and dextran production across all strains (Figures 5, 6). High-EPS producers, such as BSDF25-7 and BSDF48-3, yielded abundant dextran, correlating with elevated CFU counts but not necessarily total biomass, suggesting a loosely bound or soluble extracellular matrix. In contrast, BSDF2-3 produced modest EPS mass but high viscosity and biofilm biomass, implying a denser, possibly branched or insoluble polymeric network. Previous studies emphasize that EPS in LAB, such as *Leuconostoc,* include diverse glucans, often mixtures of dextran and levan, each influencing viscosity, adhesion, and texture differently (Jurášková et al., 2022; Du et al., 2023). The strong positive correlations observed between EPS quantity and dextran concentration confirm that dextran is a key contributor to the matrix, though not the sole determinant (Figure 7). Notably, one report in the literature indicates that *L. citreum* biofilms have relatively low protein content relative to carbohydrate (Labadie et al., 2021). Further, another report suggests that both dextran and nucleic acid are involved in *Leuconostoc* biofilm formation (Badel et al., 2008). These results suggest compositional complexity, potentially involving other glucans, proteins, or extracellular DNA, all of which may modulate biofilm structure and resistance traits (Flemming and Wingender, 2010; Torres-Rodríguez et al., 2014).

Strains such as *L. suionicum* BSDF2-3 and *L. citreum* BSDF5-1, which form resilient biofilms, pose significant challenges for cleaning protocols and may promote biofilm-associated clogging or contamination. Conversely, dextran-rich but weakly adherent strains, such as BSDF25-7, may still clog filters during sugar beet processing despite weaker adherence. Further, these types of *Leuonostoc* isolates are advantageous for controlled EPS production for food applications, such as texture enhancement, provided conditions are optimized for yield and polymer quality.

Understanding the unique biochemical traits attributable to each bacterial EPS is crucial, as this knowledge can inform utilization in biotechnology or removal in industrial settings. Gaining deeper insights into the various aspects of the biofilm matrix is vital for developing better methods to prevent and address biofilm-related problems in sugar processing facilities. To further understand the structural aspects of biofilm formation, the role of EPS structure, as well as the contributions of additional extracellular matrix components such as DNA and proteins, ought to be explored in future research. Additionally, the complex dynamics of matrix formation, structural organization, and remodeling in interspecies and interkingdom interactions in industrial biofilms must be considered (Qi et al., 2023).

This investigation underscored the critical role of EPS in *Leuconostoc* biofilm formation. However, the results also demonstrate that EPS quantity alone does not fully predict biofilm performance; rather, the growth rate, architecture, composition, and viscoelastic properties of the matrix contribute to persistence and function. While the various *Leuconostoc* strains exhibited phenotypic variation in CFUs and biofilm accumulation over time during the batch phase and continuous flow phase, future studies are needed to further investigate, more specifically, the time-dependent dynamics of biofilm formation and biomass accumulation. These dynamics are likely strain-specific adaptive mechanisms that regulate biofilm formation in *Leuconostoc* species.

## 5 Conclusion

This study demonstrates marked strain-specific variation in biofilm formation, EPS production, and growth kinetics among nine *Leuconostoc* isolates from sugar beet processing environments. Notably, matrix accumulation and biofilm resilience were not always linked to CFU counts or EPS yield, indicating functional diversity in biofilm architecture. The unique combination of high culture viscosity and low EPS production in strains, such as BSDF2-3, points to novel extracellular matrix compositions, with significant ramifications for sanitation procedures and the management of biofouling in industrial settings. Conversely, strains with high dextran output may have potential in biotechnological applications, such as food texturization. These findings highlight the importance of evaluating both structural and compositional attributes of biofilms and underscore the need for further studies on EPS diversity and regulation to guide targeted control or exploitation of *Leuconostoc* species.

## Data Availability

The original contributions presented in the study are included in the article/Supplementary material, further inquiries can be directed to the corresponding author.
