# Supplementary material for: Phenotypic variation in growth and biofilm formation of Leuconostoc spp. from sugar beet factories
Source: Front Microbiol. 2026 Jan 15;16:1745936. doi: 10.3389/fmicb.2025.1745936 (PMC12853659; doi:10.3389/fmicb.2025.1745936)
Supplement: Supplementary file 1 [file Data_Sheet_1.docx]

Supplementary Material S1

The supplementary data includes full reporting of results from viscosity analysis of the nine culture strains utilized in the study. The strains are divided here into two separate categories: “Less Viscous” (BSDF48-3, BSDF14-9, BSDF47-1, BSDF25-7, BSDF62-9, BSDF52-11) and “More Viscous” (BSDF2-3, BSDF2-6, BSDF5-1). The “Less Viscous” results are given in Table s1 , and the “More Viscous” results are given in Table s2.

The tables report the strain number, replicate number (order of analysis was random among three replicate flasks), optical density (OD_600_), and measured viscosity with associated speed and torque (T) percentage as read from the viscometer instrument. The viscosity measurement reported in the main text is the measured viscosity at the lowest instrument speed setting, where the measured torque output exceeded a value of 10%; instrument manufacturer recommendation is to only use viscosity readings with associated torque values between 10-100%.

Finally, Table s3 provides statistical analyses of the results presented in Table s1 and Table s2, along with some associated discussion.

**Table s1.** Viscosity measurement results for “Less Viscous” flask cultures.

| Strain no. | Replicate Flask no. | OD600 | Speed (1)  Speed (2)  Speed (3) | T% (1)  T% (2)  T% (3) | Visc. (1)  Visc. (2)  Visc. (3) | Remark |
| --- | --- | --- | --- | --- | --- | --- |
| 48-3 | 1 | 2.70 | 5 RPM  10 RPM  20 RPM | 8.6  17.0  34.3 | 10.3 cP  10.2 cP  10.4 cP | The selected viscosity readings among these replicates are 10.2 cP, 11.3 cP, and 11.0 cP. |
| 48-3 | 2 | 2.88 | 5 RPM  10 RPM  20 RPM | 9.5  18.9  37.8 | 11.4 cP  11.3 cP  11.3 cP |  |
| 48-3 | 3 | 2.78 | 5 RPM  10 RPM  20 RPM | 9.2  18.4  37.2 | 10.9 cP  11.0 cP  11.2 cP |  |
| 14-9 | 1 | 2.38 | 10 RPM  20 RPM  50 RPM | 5.2  10.5  26.4 | 3.12 cP  3.15 cP  3.17 cP | The selected viscosity readings among these replicates are 3.15 cP, 3.09 cP, and 3.00 cP. |
| 14-9 | 2 | 2.39 | 10 RPM  20 RPM  50 RPM | 5.1  10.3  26.0 | 3.06 cP  3.09 cP  3.12 cP |  |
| 14-9 | 3 | 2.48 | 10 RPM  20 RPM  50 RPM | 5.0  10.0  25.6 | 3.00 cP  3.00 cP  3.07 cP |  |
| 47-1 | 1 | 2.47 | 10 RPM  20 RPM  50 RPM | 5.0  9.9  24.7 | 3.06 cP  2.97 cP  2.96 cP | The selected viscosity readings among these replicates are 2.96 cP, 3.09 cP, and 3.03 cP. |
| 47-1 | 2 | 2.45 | 10 RPM  20 RPM  50 RPM | 5.2  10.3  26.2 | 3.12 cP  3.09 cP  3.14 cP |  |
| 47-1 | 3 | 2.48 | 10 RPM  20 RPM  50 RPM | 5.2  10.1  25.4 | 3.12 cP  3.03 cP  3.05 cP |  |
| 25-7 | 1 | 2.67 | 5 RPM  10 RPM  20 RPM | 9.3  18.7  37.6 | 11.2 cP  11.2 cP  11.3 cP | The selected viscosity readings among these replicates are 11.2 cP, 10.6 cP, and 10.5 cP. |
| 25-7 | 2 | 2.67 | 5 RPM  10 RPM  20 RPM | 8.8  17.7  35.8 | 10.6 cP  10.6 cP  10.7 cP |  |
| 25-7 | 3 | 2.72 | 5 RPM  10 RPM  20 RPM | 8.9  17.5  35.8 | 10.7 cP  10.5 cP  10.7 cP |  |
| 62-9 | 1 | 2.02 | 10 RPM  20 RPM  50 RPM | 9.7  19.3  47.5 | 5.82 cP  5.79 cP  5.70 cP | The selected viscosity readings among these replicates are 5.79 cP, 6.12 cP, and 4.98 cP. |
| 62-9 | 2 | 2.01 | 10 RPM  20 RPM  50 RPM | 10.2  20.3  50.2 | 6.12 cP  6.09 cP  6.02 cP |  |
| 62-9 | 3 | 2.05 | 10 RPM  20 RPM  50 RPM | 8.4  16.7  41.5 | 5.04 cP  4.98 cP  4.98 cP |  |
| 52-11 | 1 | 2.78 | 10 RPM  20 RPM  50 RPM | 5.8  11.5  29.1 | 3.48 cP  3.45 cP  3.49 cP | The selected viscosity readings among these replicates are 3.45 cP, 3.42 cP, and 3.39 cP. |
| 52-11 | 2 | 2.90 | 10 RPM  20 RPM  50 RPM | 5.7  11.4  28.4 | 3.42 cP  3.42 cP  3.41 cP |  |
| 52-11 | 3 | 2.82 | 10 RPM  20 RPM  50 RPM | 5.7  11.3  28.4 | 3.42 cP  3.39 cP  3.41 cP |  |

**Table s2.** Viscosity measurement results for “More Viscous” flask cultures.

| Strain no. | Replicate Flask no. | OD600 | Speed (1)  Speed (2)  Speed (3) | T% (1)  T% (2)  T% (3) | Visc. (1)  Visc. (2)  Visc. (3) | Remark |
| --- | --- | --- | --- | --- | --- | --- |
| 2-3 | 1 | 7.94 | 0.5 RPM  --  -- | 97.0  --  -- | 1164 cP  --  -- | The selected viscosity readings among these replicates are 1164 cP, 1051cP, and 1070 cP. Because of the high viscosity of the sample, it was not possible to successfully collect a measurement at any speed greater than 0.5 RPM. |
| 2-3 | 2 | 6.72 | 0.5 RPM  --  -- | 87.6  --  -- | 1051 cP  --  -- |  |
| 2-3 | 3 | 6.64 | 0.5 RPM  --  -- | 89.2  --  -- | 1070 cP  --  -- |  |
| 2-6 | 1 | 6.90 | 0.5 RPM  --  -- | 100  --  -- | 1200 cP  --  -- | The viscosity reading used for all three replicates for this sample is 1200 cP. In each case, the instrument torque was maxed out (>100%) at the lowest speed (0.5 RPM) because of very high sample viscosity. Therefore, this value represents an approximation of the viscosity, but it is not the true viscosity of the sample. The true sample viscosity is higher and would require a different instrument configuration for more accurate analysis. |
| 2-6 | 2 | 5.95 | 0.5 RPM  --  -- | 100  --  -- | 1200 cP  --  -- |  |
| 2-6 | 3 | 5.91 | 0.5 RPM  --  -- | 100  --  -- | 1200 cP  --  -- |  |
| 5-1 | 1 | 5.45 | 0.5 RPM  1 RPM  -- | 39.6  62.2  -- | 475.1 cP  371.9 cP  -- | The selected viscosity readings among these replicates are 475.1 cP, 595.1 cP, and 848.2 cP. Because of the high viscosity of the sample, it was not possible to successfully collected a measurement at speed 1 RPM for the third replicate flask, although the previous two replicates did work at 1 RPM. |
| 5-1 | 2 | 5.32 | 0.5 RPM  1 RPM  -- | 49.6  72.5  -- | 595.1 cP  434.9 cP  -- |  |
| 5-1 | 3 | 6.06 | 0.5 RPM  1 RPM  -- | 70.7  100  -- | 848.2 cP  600 cP  -- |  |

Statistical analysis was conducted on viscosity data for all samples, using log10 transformed data. ANOVA was performed followed by pair-wise t-tests between all 9 strains (where α = 0.05). The log-transformed data was found to satisfy the homogeneity of variance assumption (Levene test) and the normality assumption (Jarque-Bera test), although it did not satisfy the normality assumption for Shapiro-Wilk and Kolmogorov-Smirnov tests. Nonetheless, ANOVA was conducted with consideration to the fact that ANOVA is generally considered to be robust against violation of the normality assumption. The associated p-value (approx. 1e-24) for ANOVA was found to be significant (p < 0.05). Results from statistical analysis are given in Table 3, where viscosity is reported as-measured and as log-transformed values.

**Table s3.** Statistical analysis of viscosity results.

| Strain | Source | ID | Measured viscosity ^†^ | Log-transformed viscosity ^†^ ‡ |
| --- | --- | --- | --- | --- |
| 47-1 | Juice | *L. suionicum* | 3.0 ± 0.1 cP | 0.48 ± 0.01 [a] |
| 14-9 | Biofilm | *L. suionicum* | 3.1 ± 0.1 cP | 0.49 ± 0.01 [a] |
| 52-11 | Juice | *L. suionicum* | 3.4 ± 0.0 cP | 0.53 ± 0.00 [b] |
| 62-9 | Juice | *L. mesenteroides* | 5.6 ± 0.6 cP | 0.75 ± 0.05 [c] |
| 25-7 | Biofilm | *L. suionicum* | 10.8 ± 0.4 cP | 1.03 ± 0.02 [d] |
| 48-3 | Juice | *L. suionicum* | 10.8 ± 0.6 cP | 1.03 ± 0.02 [d] |
| 5-1 | Biofilm | *L. citreum* | 639 ± 190 cP | 2.79 ± 0.13 [e] |
| 2-3 | Biofilm | *L. suionicum* | 1095 ± 61 cP | 3.04 ± 0.02 [f] |
| 2-6 | Biofilm | *L. suionicum* | 1200 ± 0 cP ^§^ | 3.08 ± 0.00 [g] |

† Reported values are mean ± standard deviation of measurements from three replicate culture flasks.

‡ Letters in brackets following log-transformed values denote statistically significant differences.

§ See remarks in Table s2 for additional explanation of viscosity measurement for BSDF2-6.

**
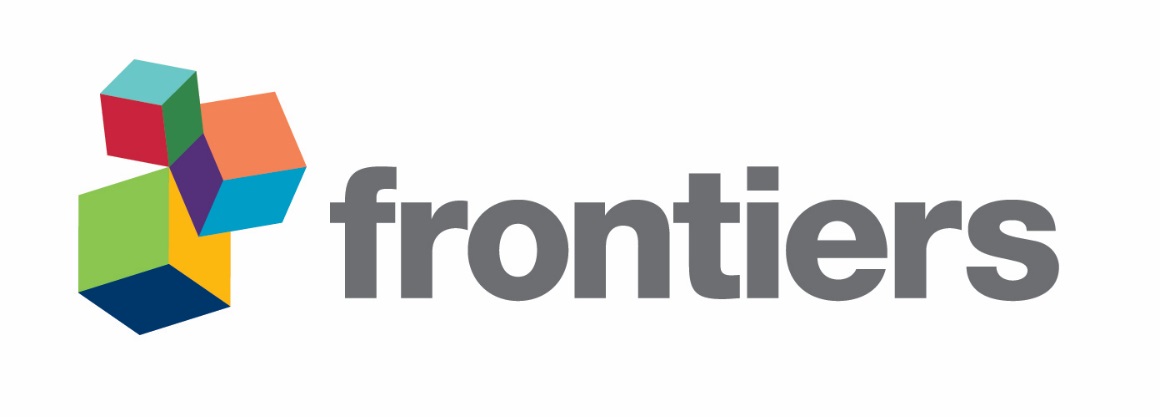
**
