# Supplementary material for: Phenotypic variation in growth and biofilm formation of Leuconostoc spp. from sugar beet factories
Source: Front Microbiol. 2026 Jan 15;16:1745936. doi: 10.3389/fmicb.2025.1745936 (PMC12853659; doi:10.3389/fmicb.2025.1745936)
Supplement: Supplementary file 2 [file Data_Sheet_2.docx]

**Supplementary S2:** Table showing biofilm-forming capacity of all nine *Leuconostoc* isolates

|  |  | **biofilm-forming capacity of bacterial strains** | | |  |
| --- | --- | --- | --- | --- | --- |
|  |  |  |  |  |  |
|  | Average CFU/cm2 | Average biomass (g) | Average CFU | biomass/CFU | **pg/CFU** |
| **Strain** |  |  |  |  |  |
| **BSDF 2-3** | 4.25E+07 | 0.1505 | 7.98E+08 | 1.89E-10 | 188.71 |
| **BSDF2-6** | 1.50E+07 | 0.021 | 2.81E+08 | 7.48E-11 | 74.75 |
| **BSDF62-9** | 8.99E+07 | 0.009 | 1.68E+09 | 5.34E-12 | 5.34 |
| **BSDF47-1** | 3.33E+08 | 0.0045 | 6.25E+09 | 7.20E-13 | 0.72 |
| **BSDF48-3** | 5.21E+08 | 0.051 | 9.77E+09 | 5.22E-12 | 5.22 |
| **BSDF14-9** | 2.60E+08 | 0.001 | 4.87E+09 | 2.05E-13 | 0.21 |
| **BSDF52-11** | 4.54E+08 | 0.008 | 8.51E+09 | 9.40E-13 | 0.94 |
| **BSDF5-1** | 5.96E+07 | 0.02 | 1.12E+09 | 1.79E-11 | 17.89 |
| **BSDF25-7** | 5.79E+08 | 0.0705 | 1.09E+10 | 6.50E-12 | 6.50 |

**
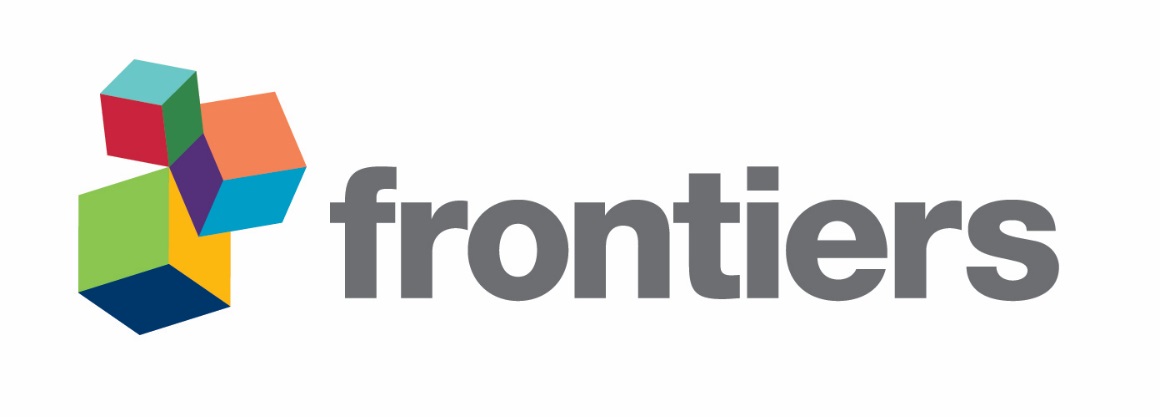
**
