# Supplementary material for: Phenotypic variation in growth and biofilm formation of Leuconostoc spp. from sugar beet factories
Source: Front Microbiol. 2026 Jan 15;16:1745936. doi: 10.3389/fmicb.2025.1745936 (PMC12853659; doi:10.3389/fmicb.2025.1745936)
Supplement: Supplementary file 3 [file Data_Sheet_3.docx]

**Supplementary S3:** Scanning electron microscope images of 48-hour biofilm from adhesion batch phase experiments with 5000x and 2000x magnifications.


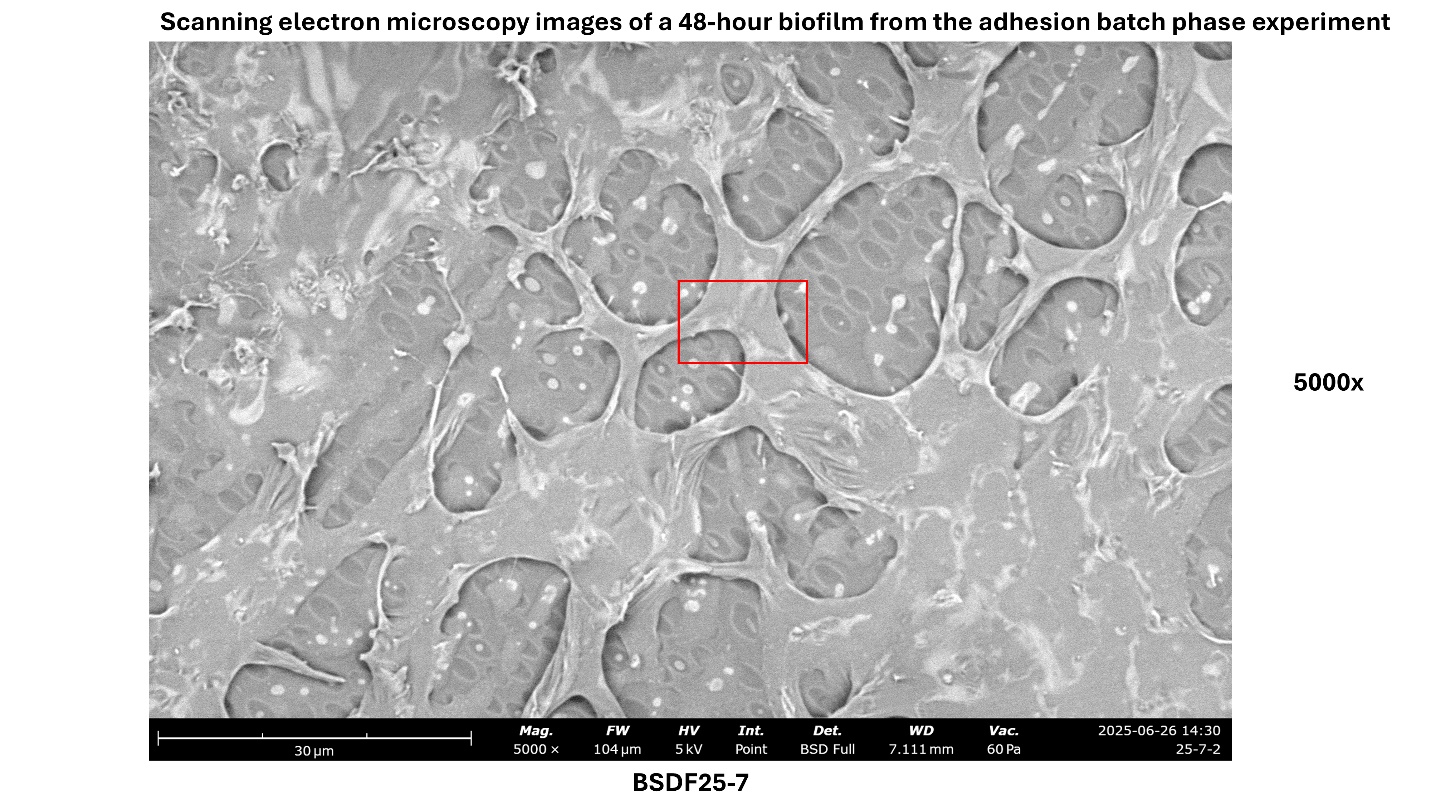

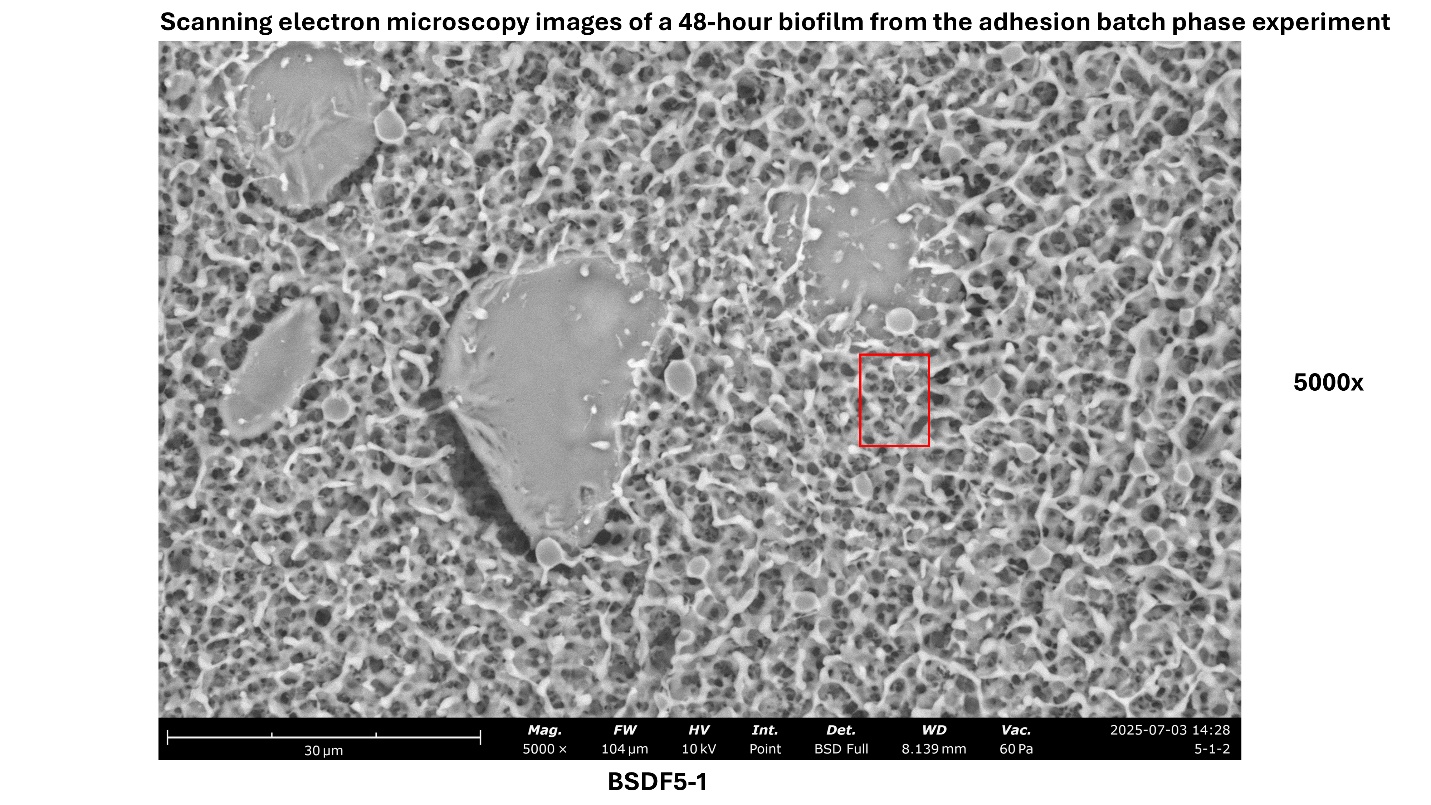

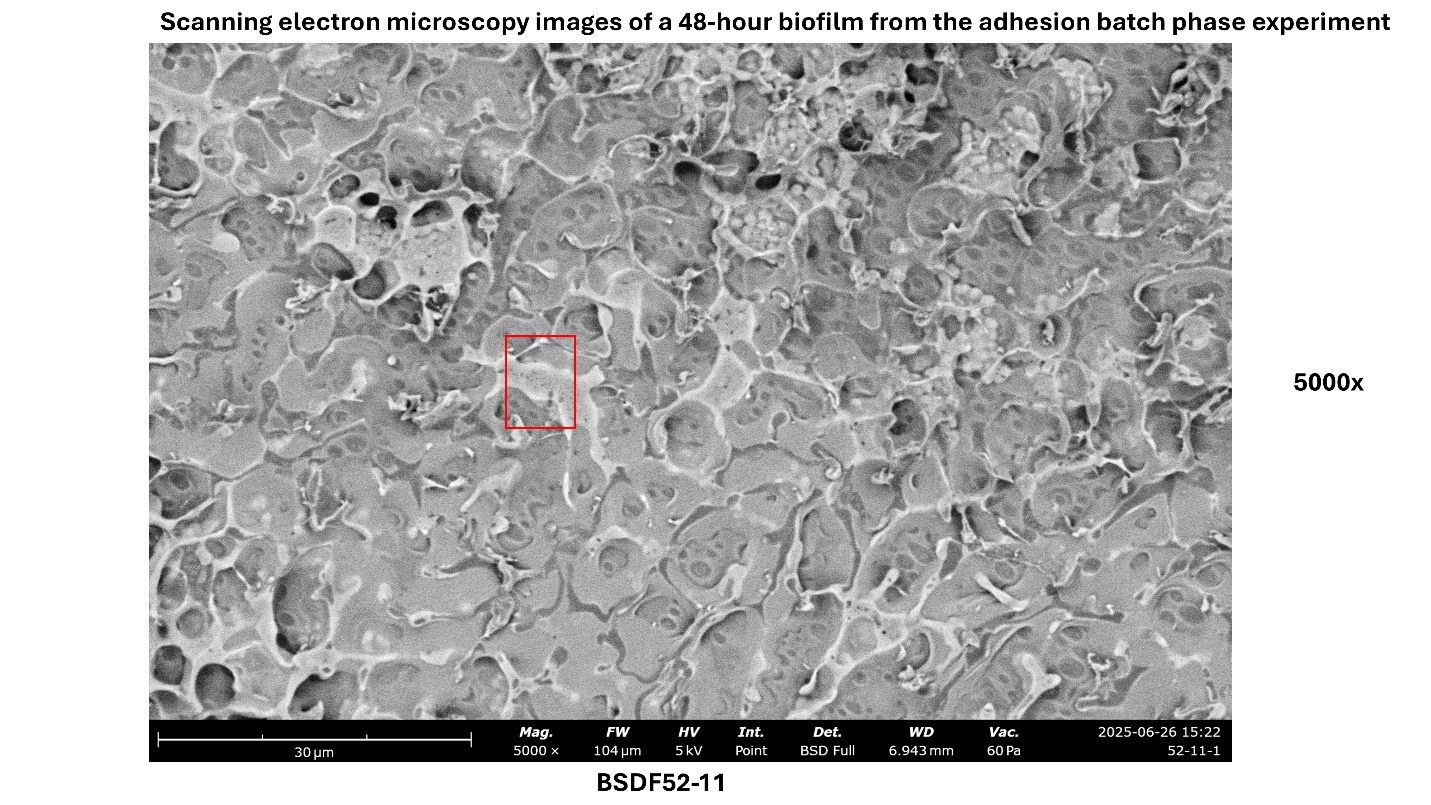

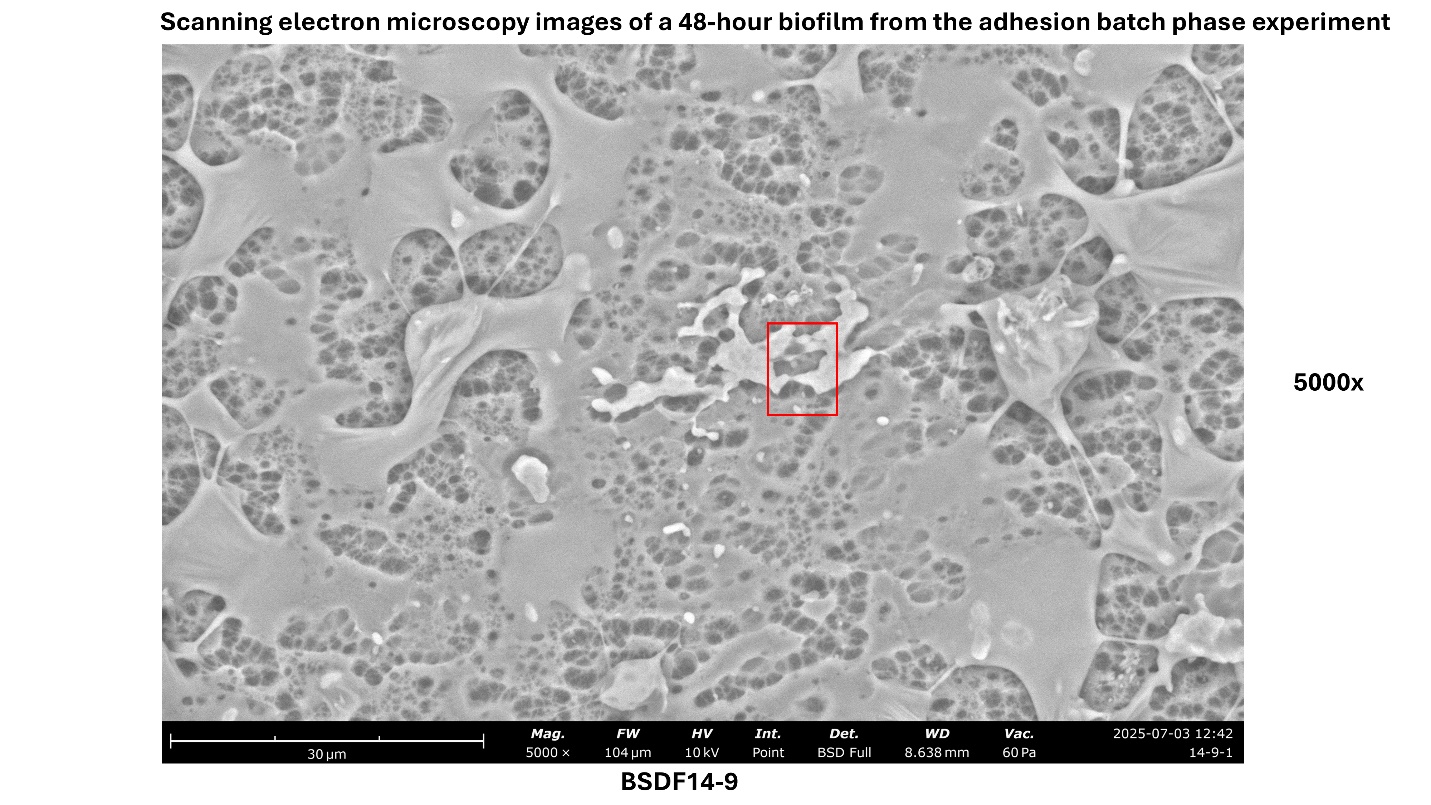

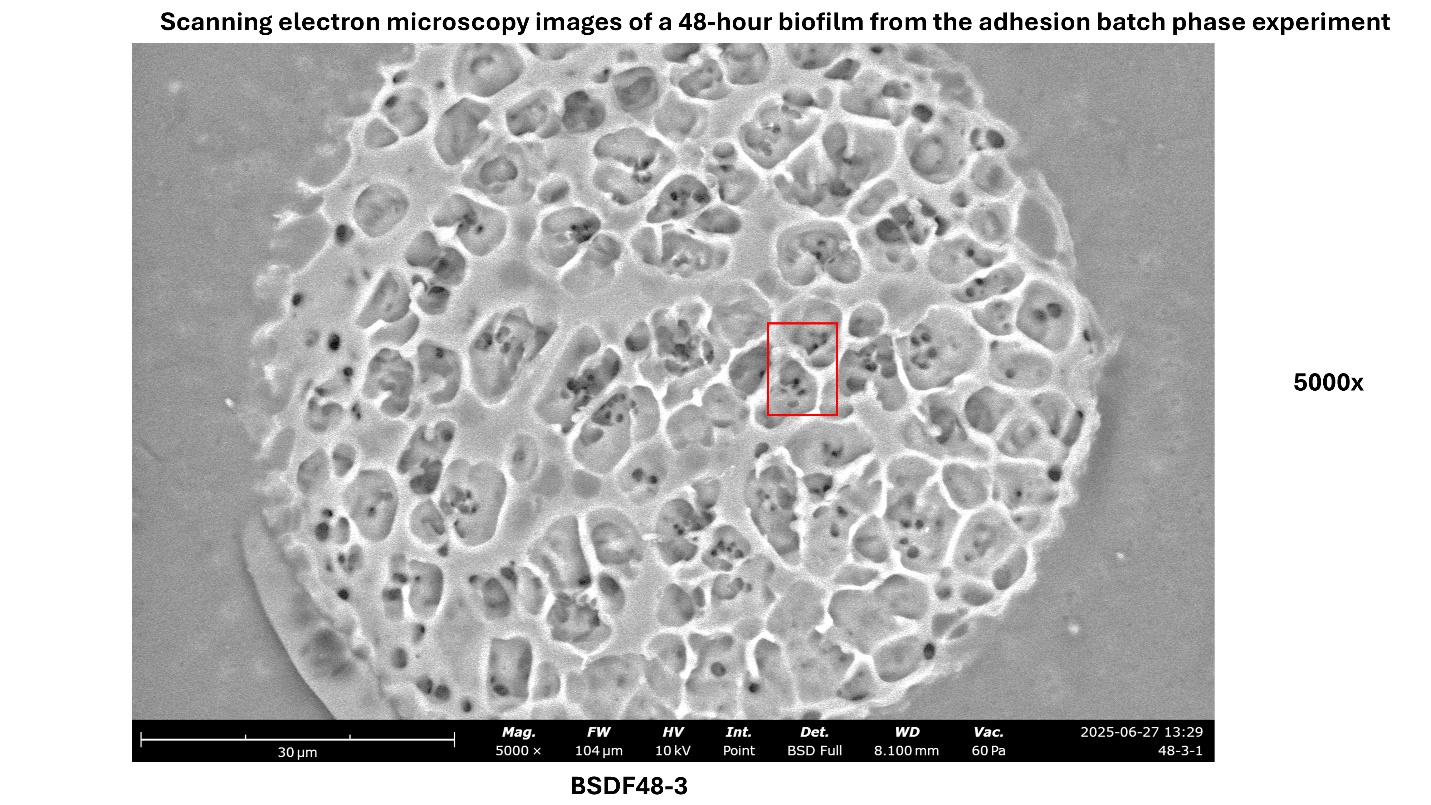

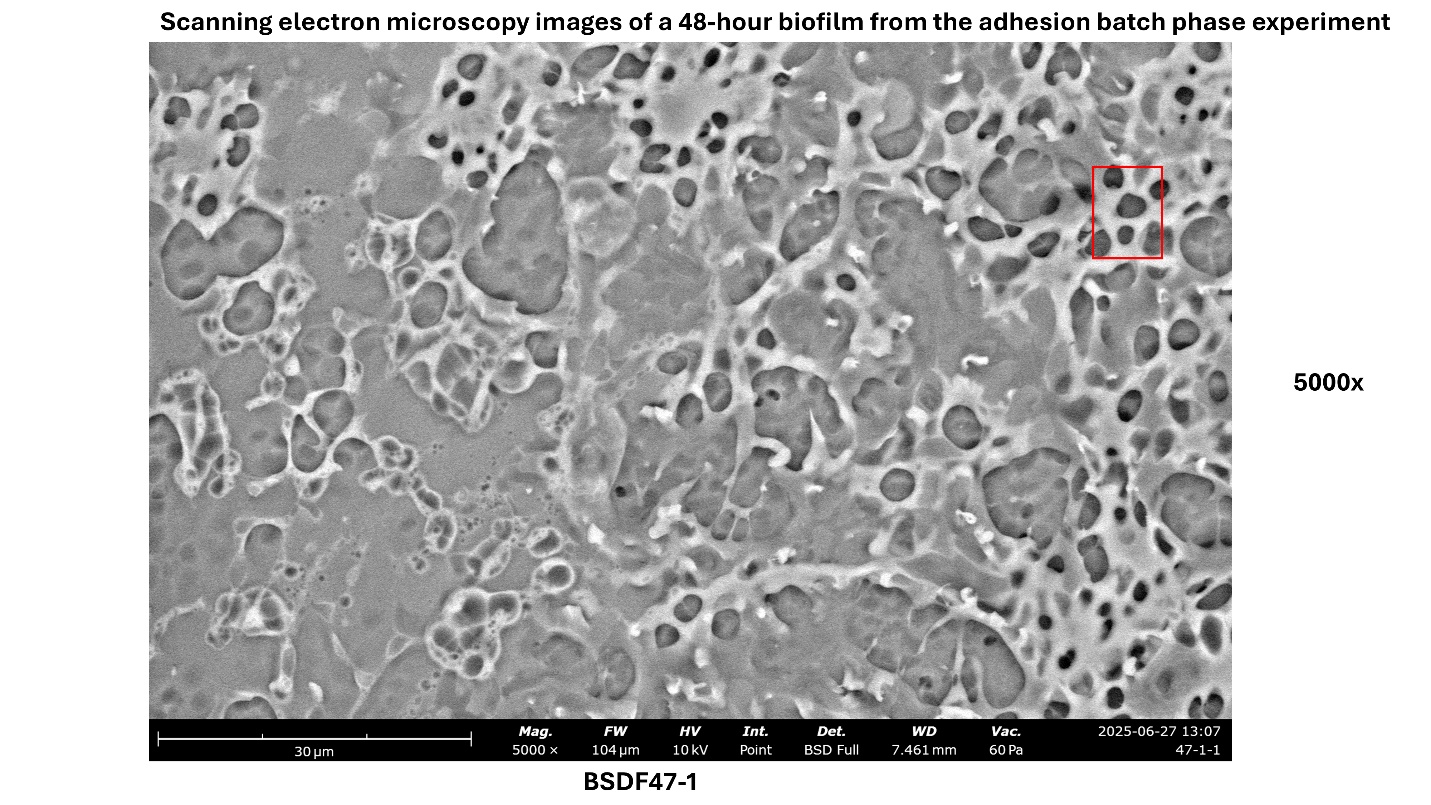

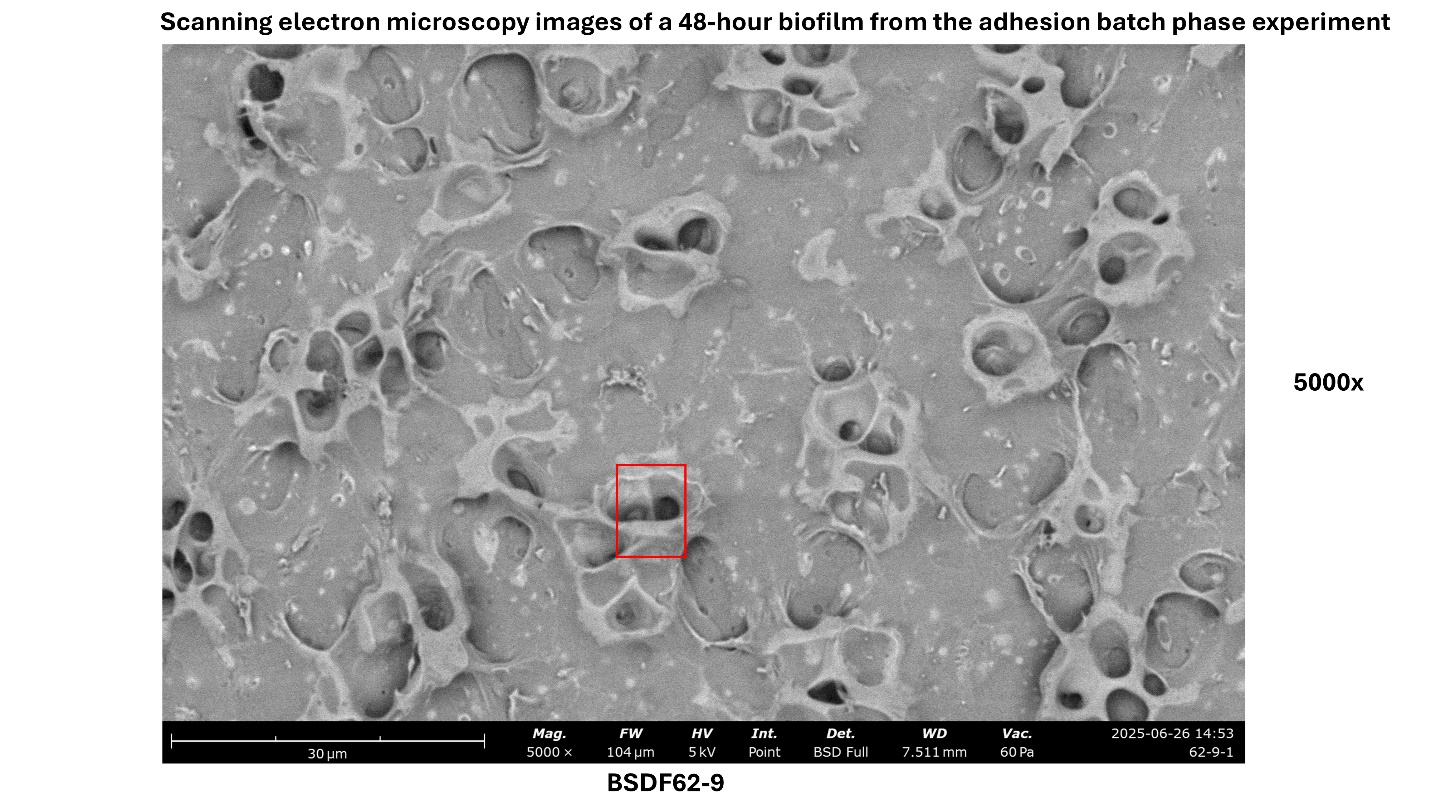

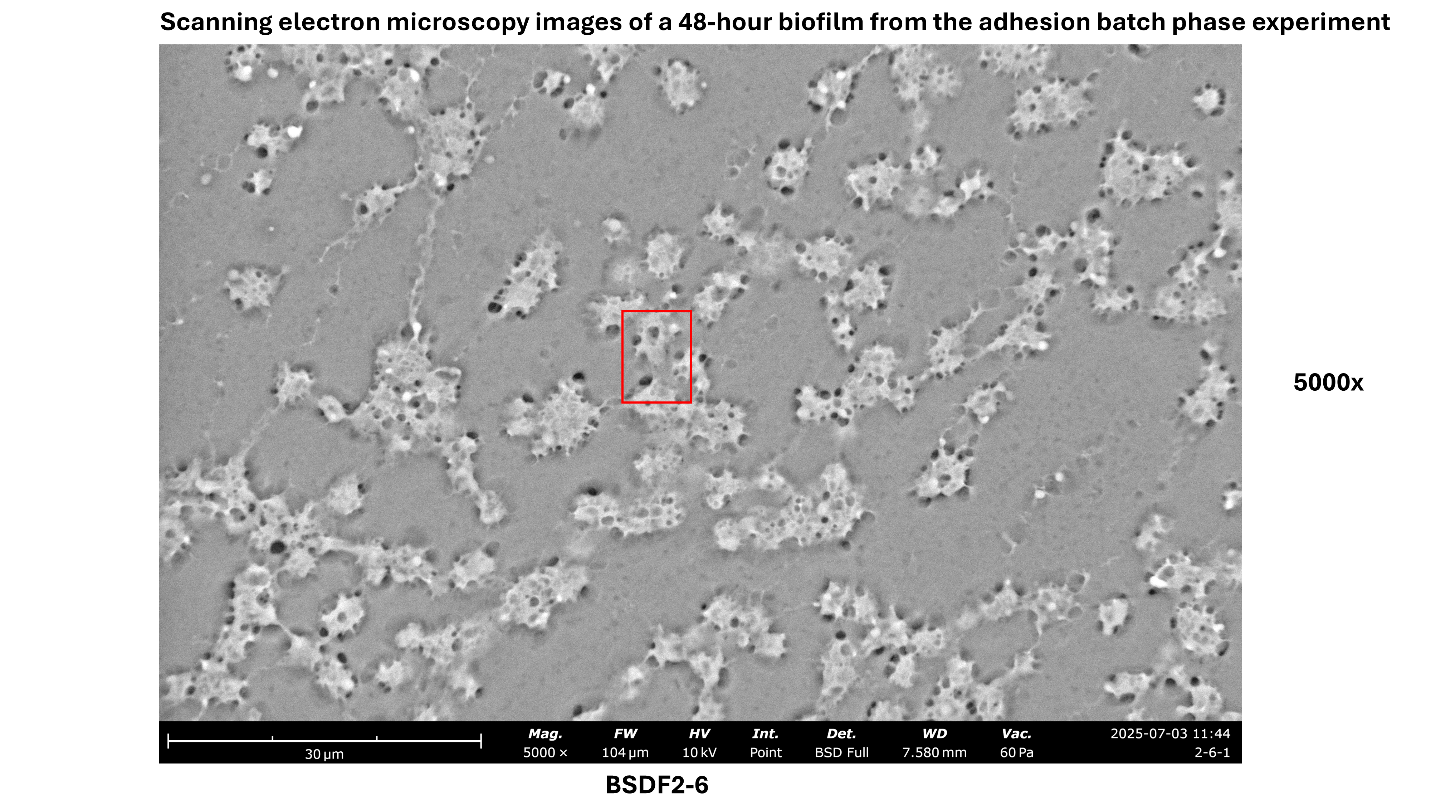

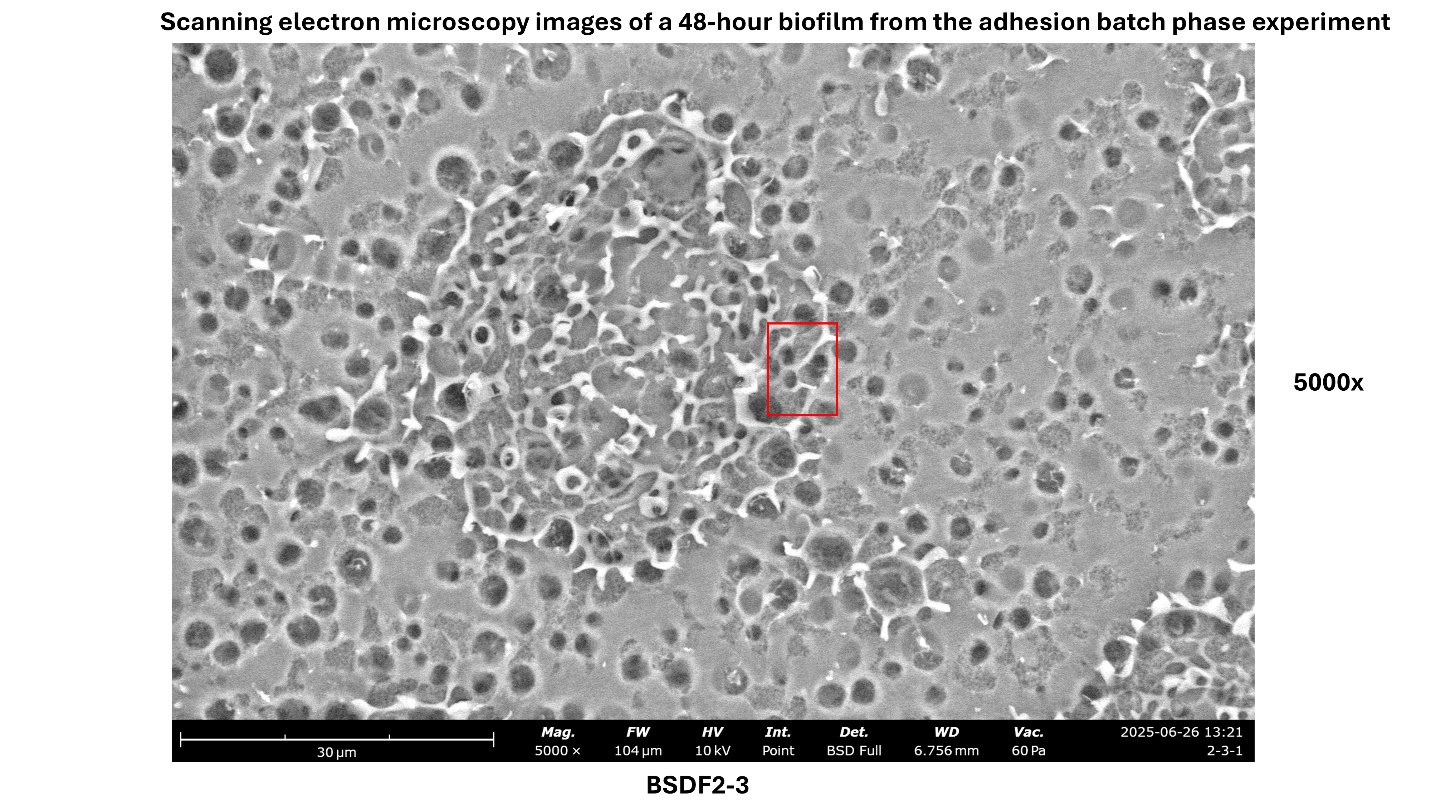

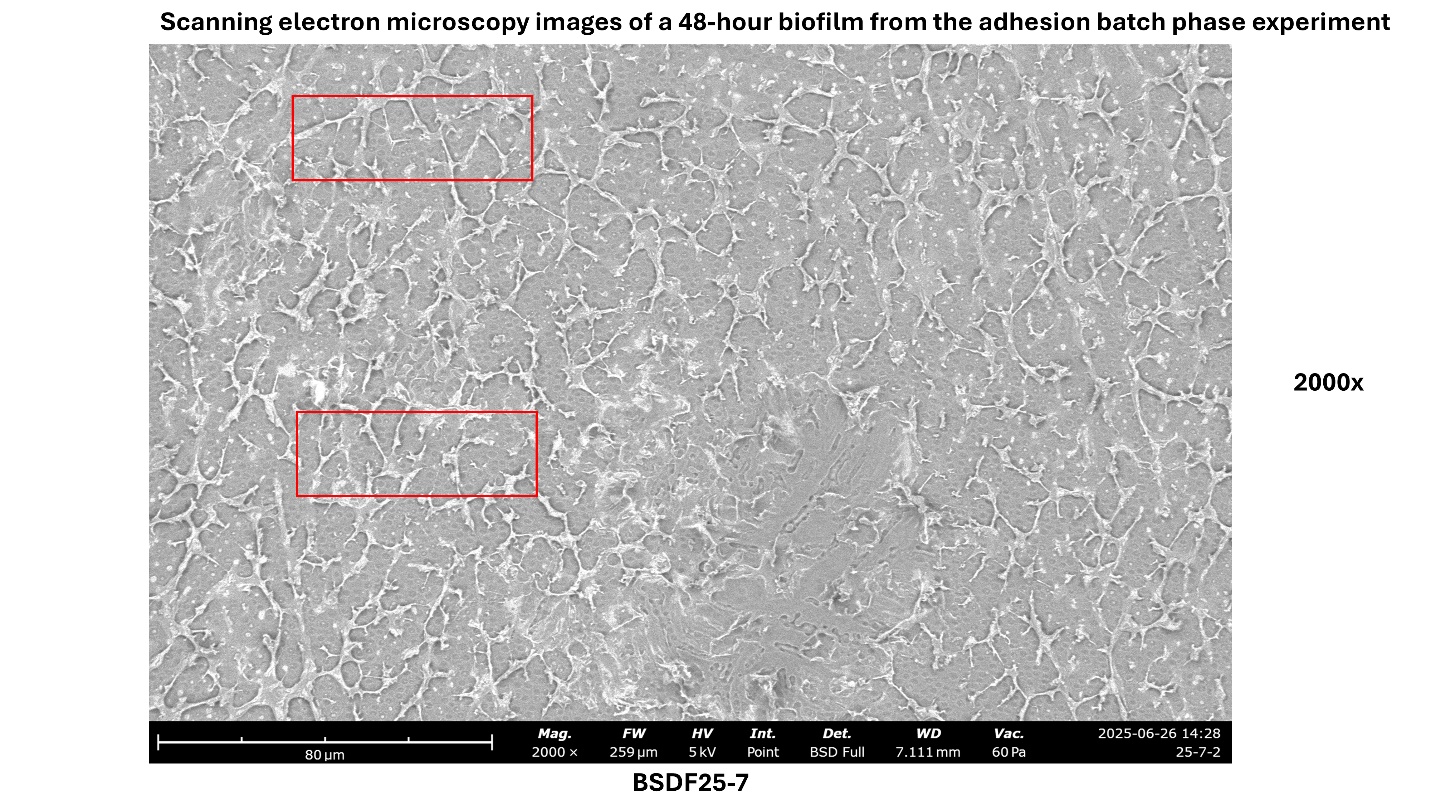

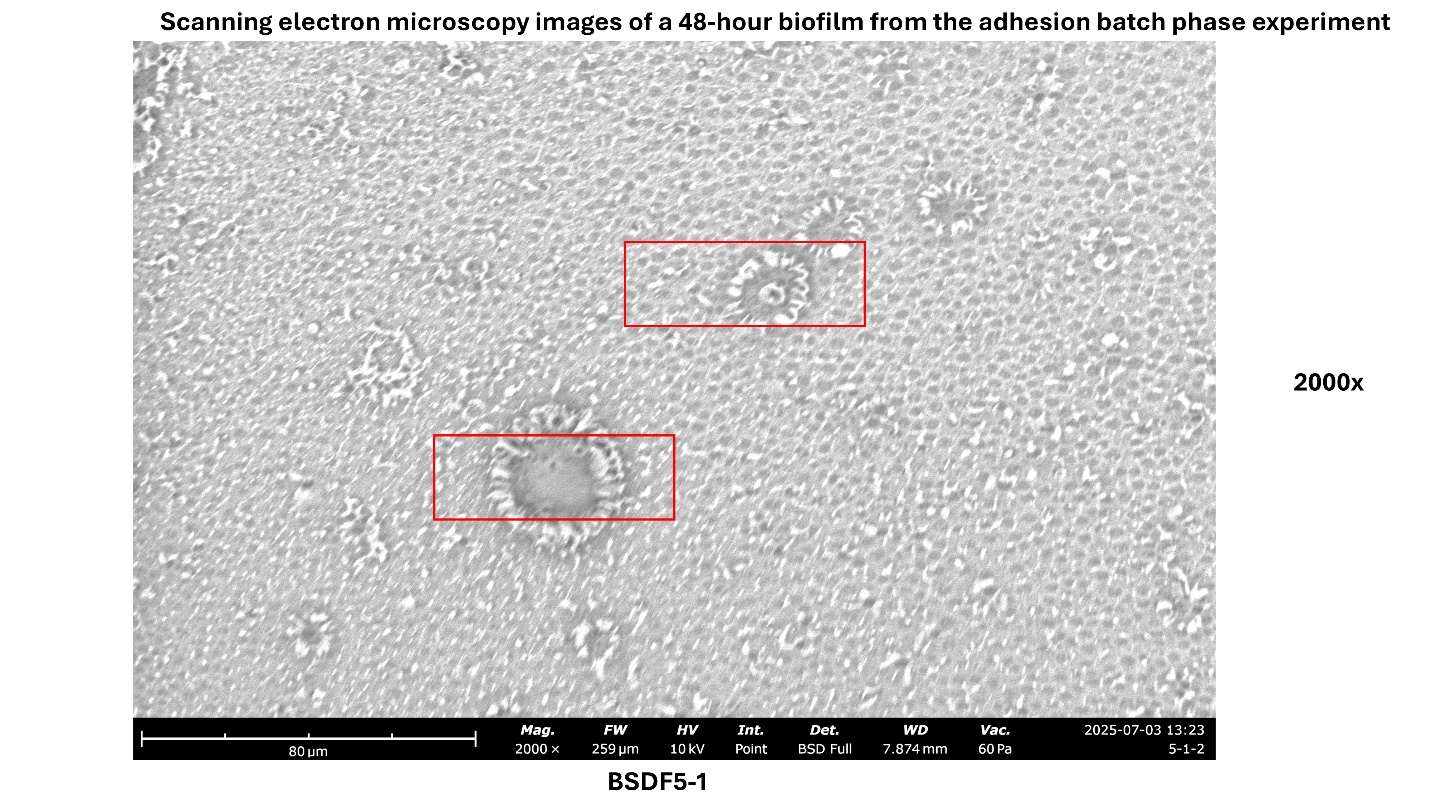

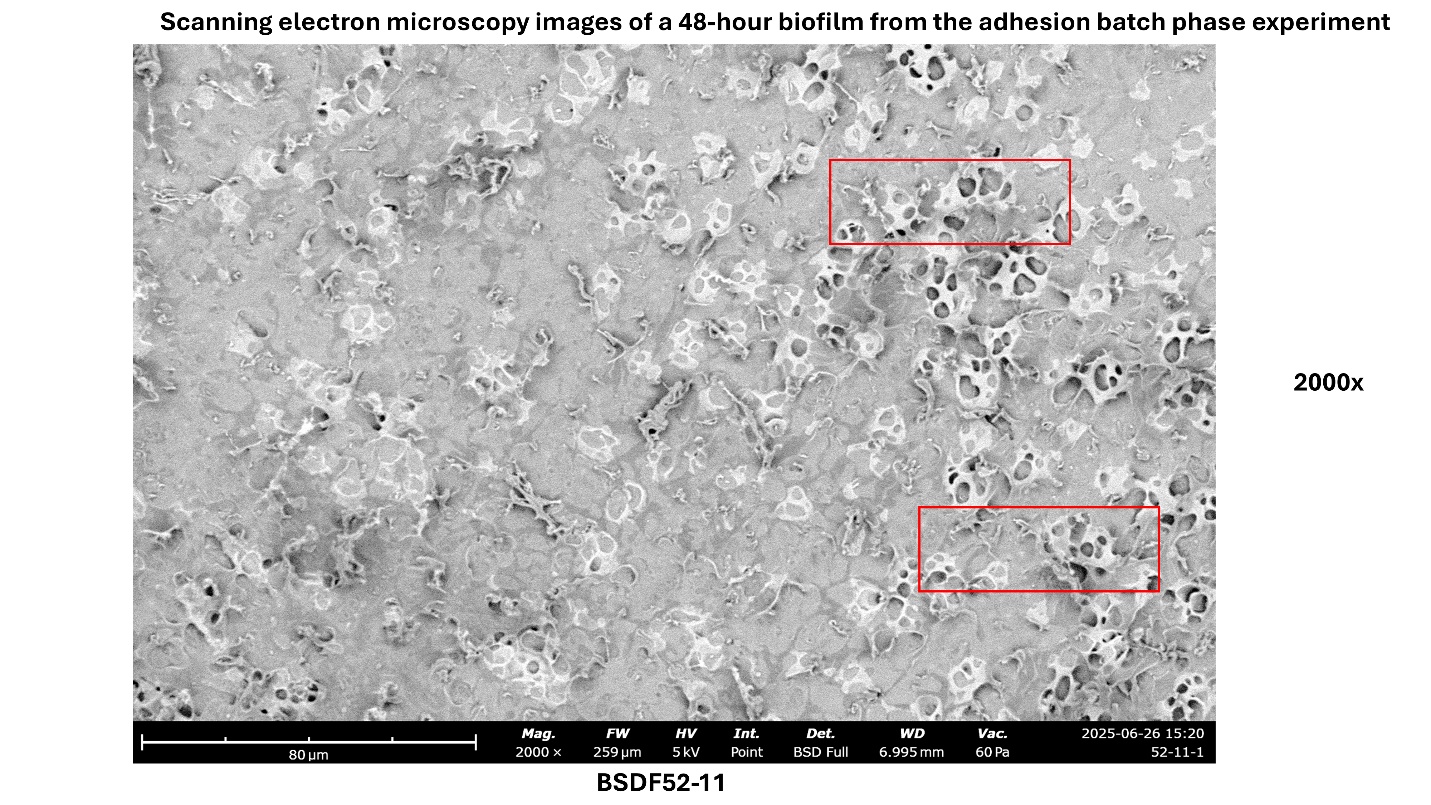

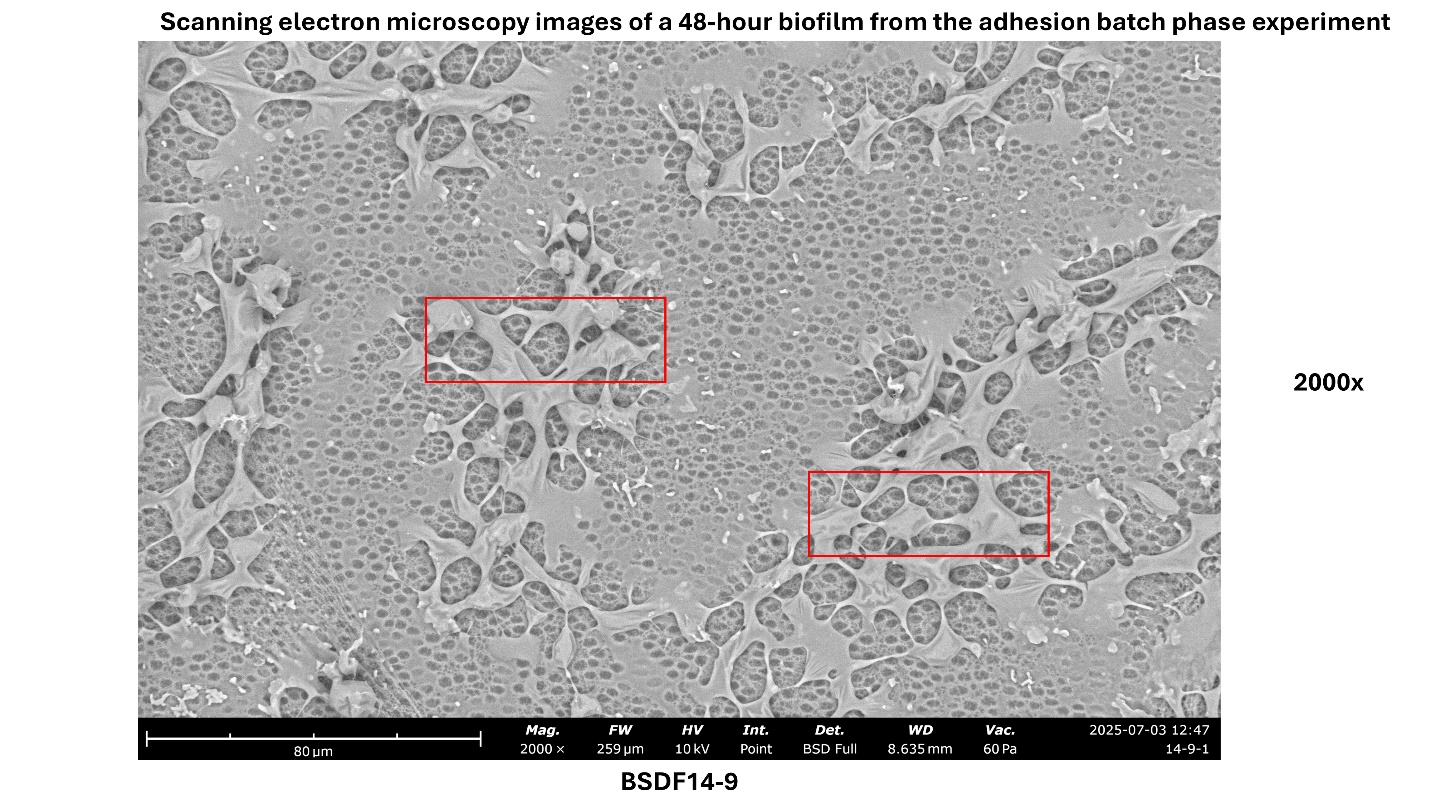

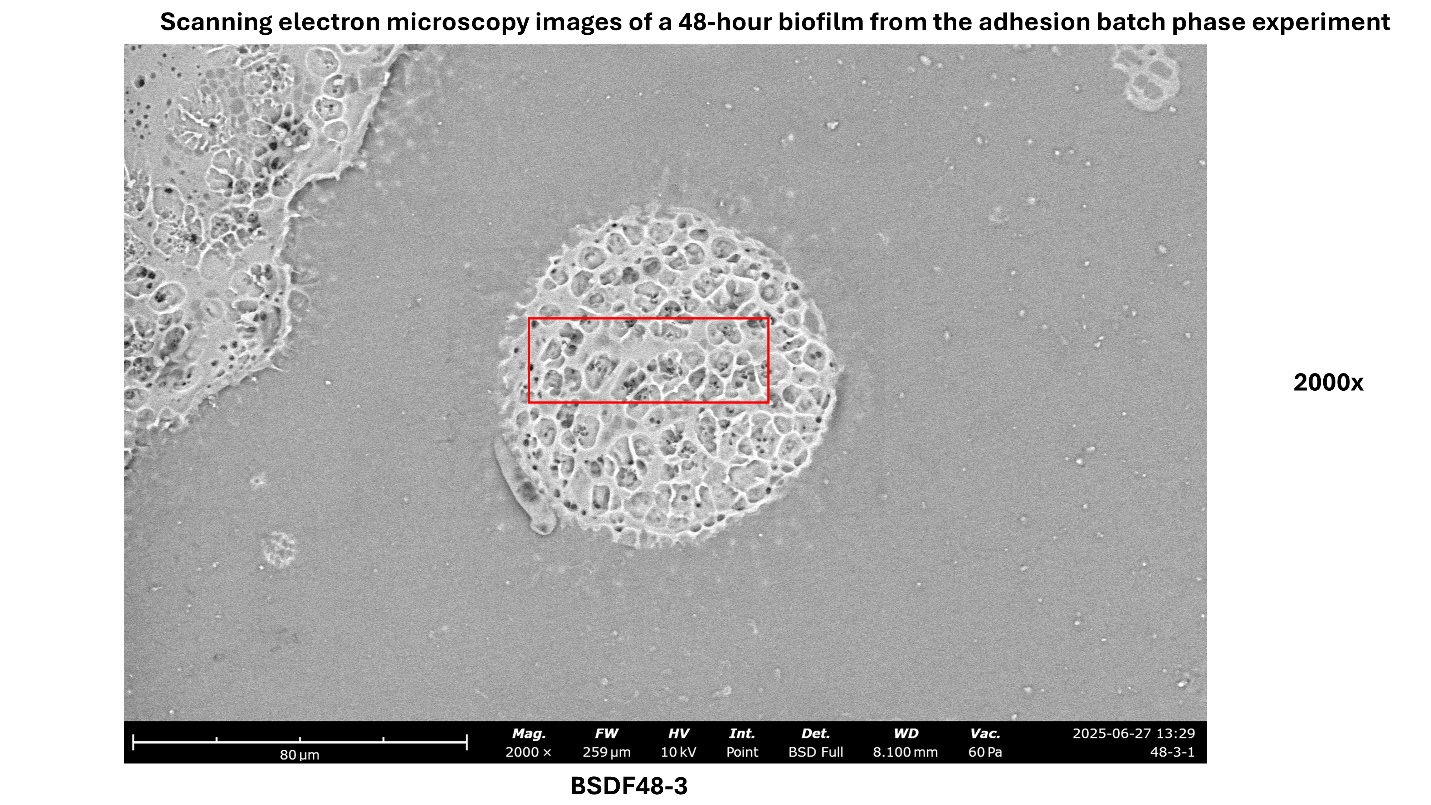

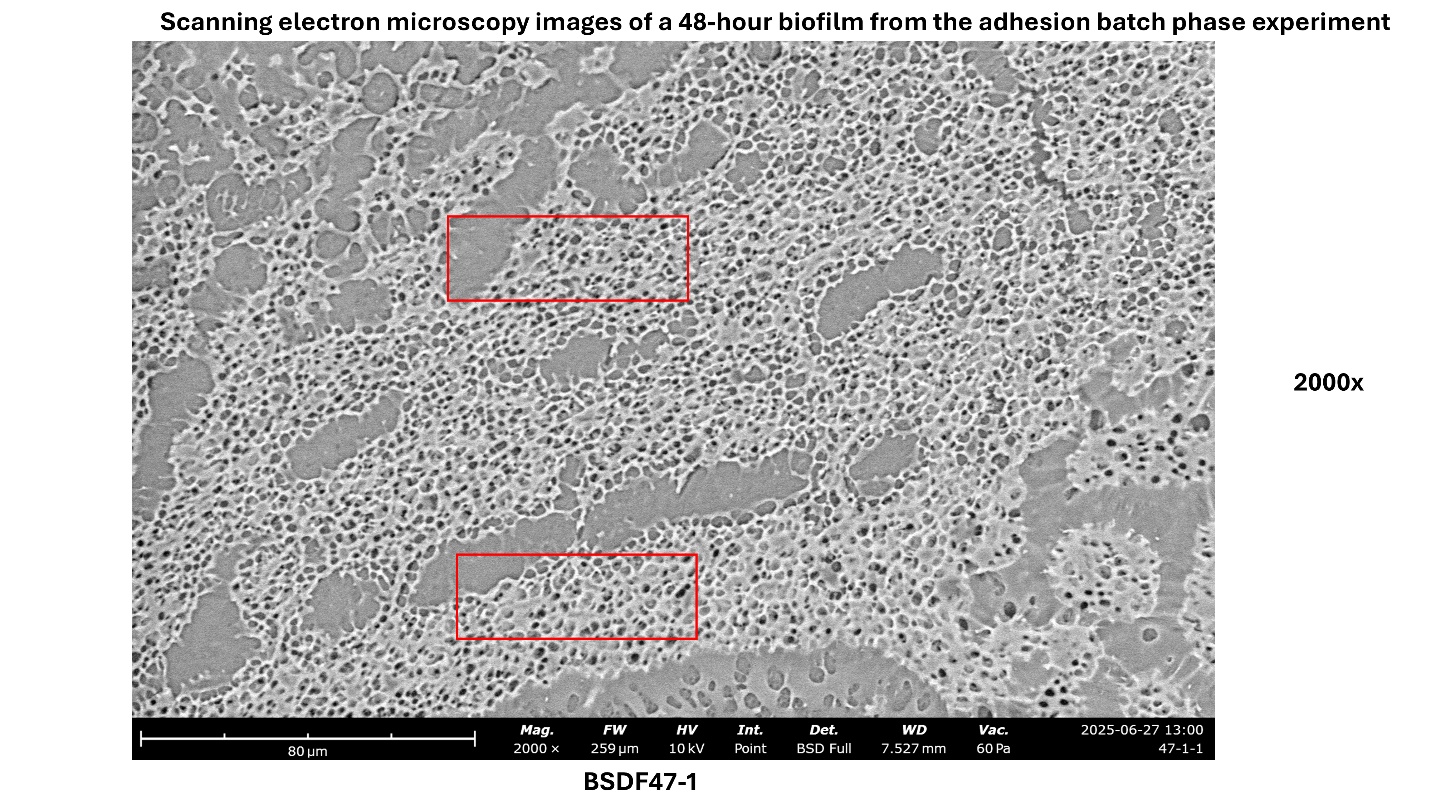

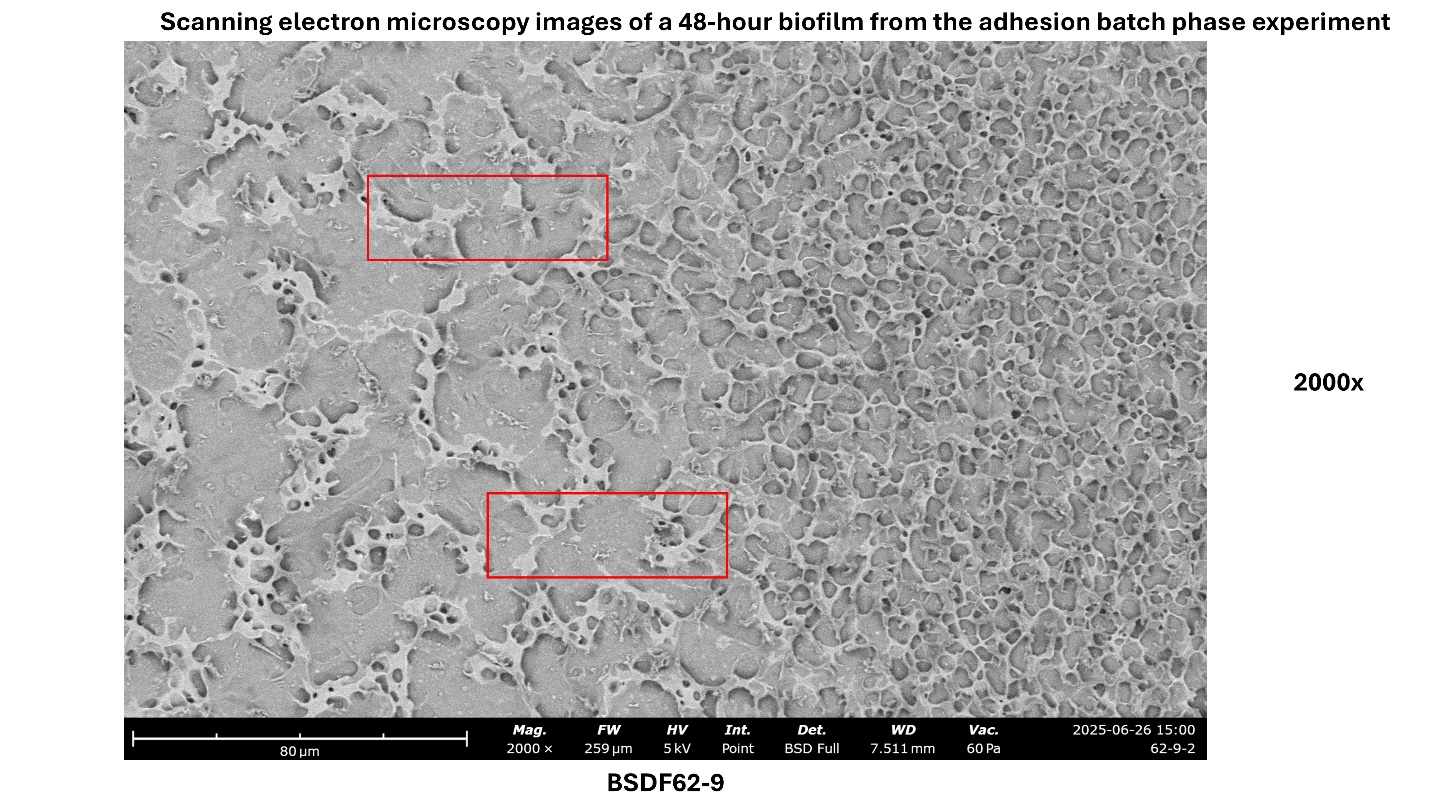

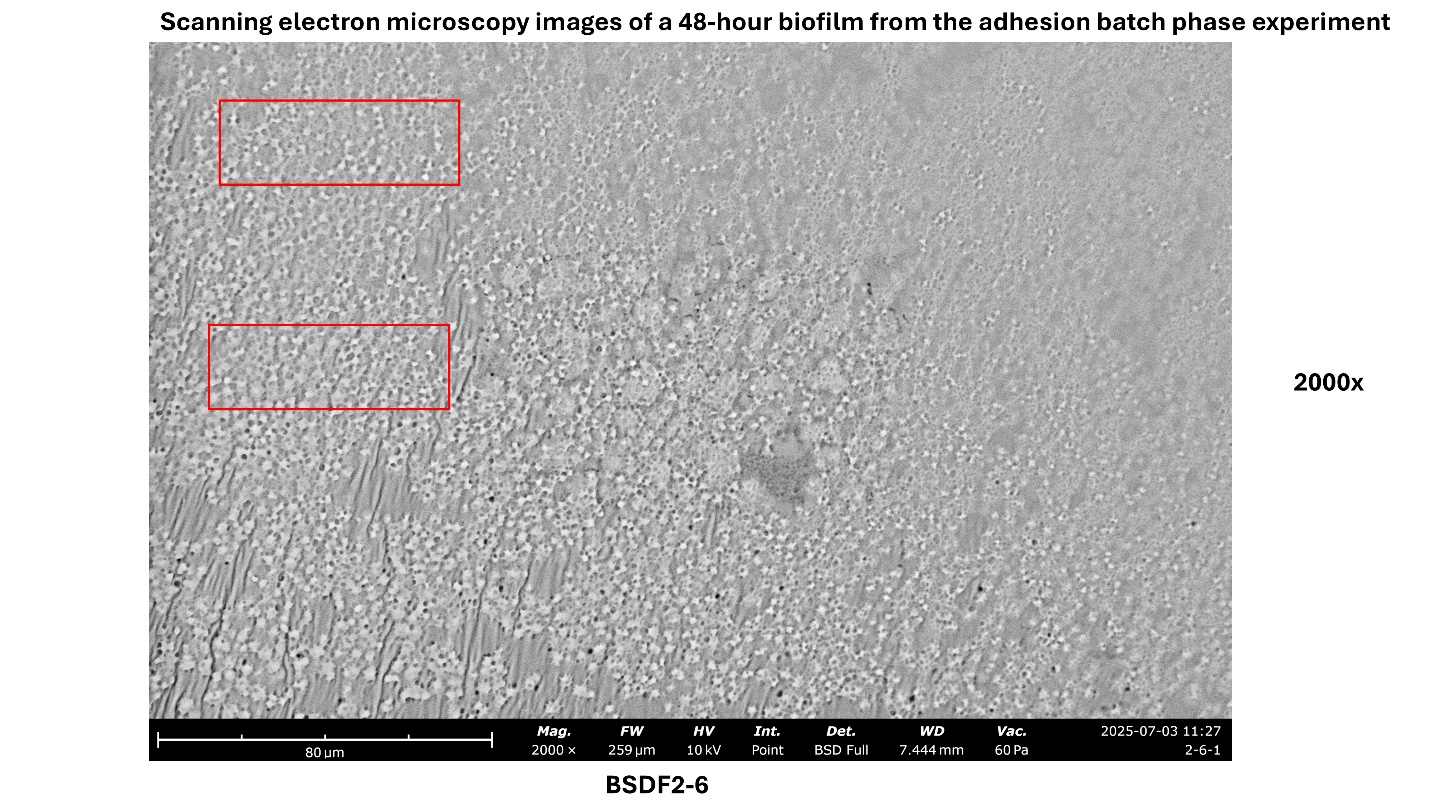

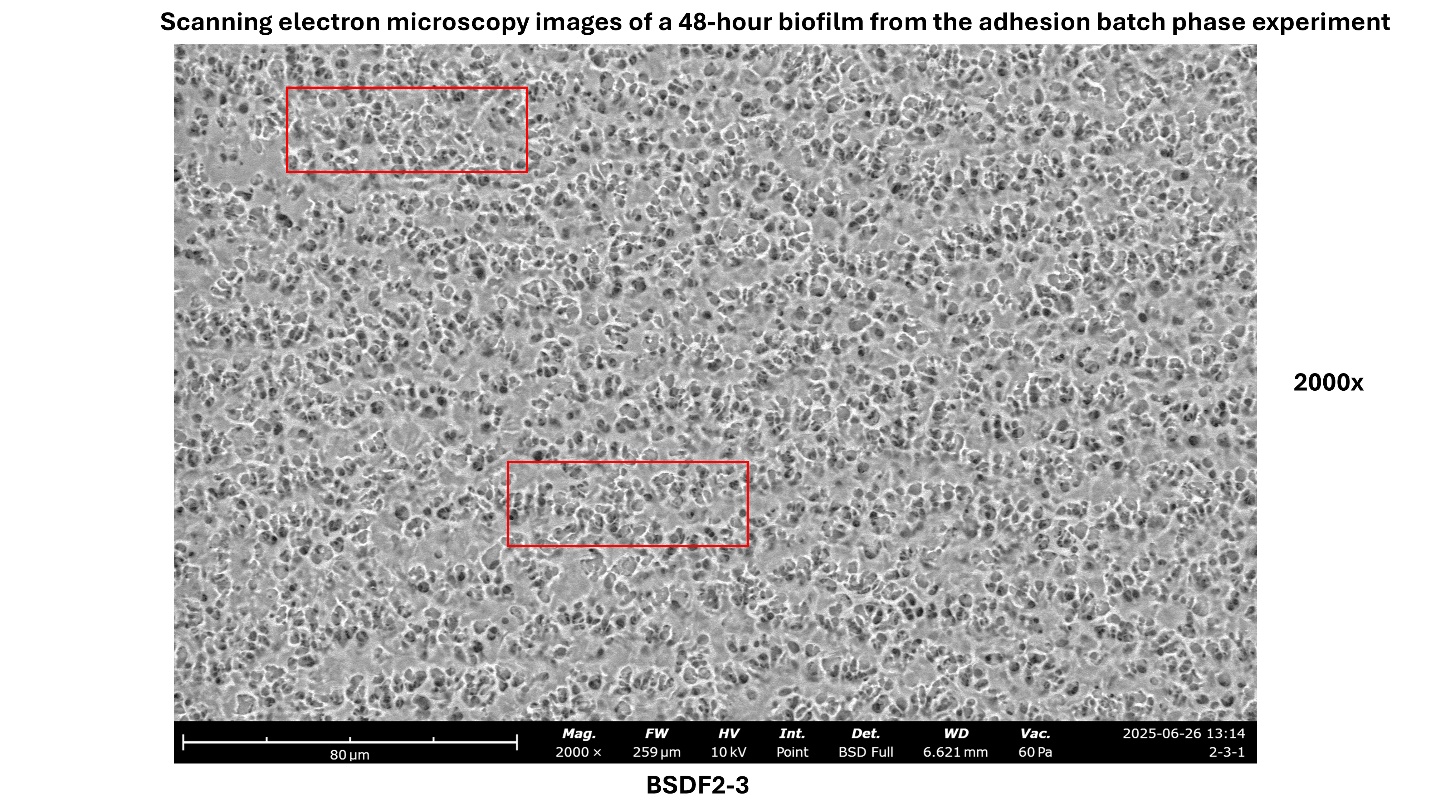


**
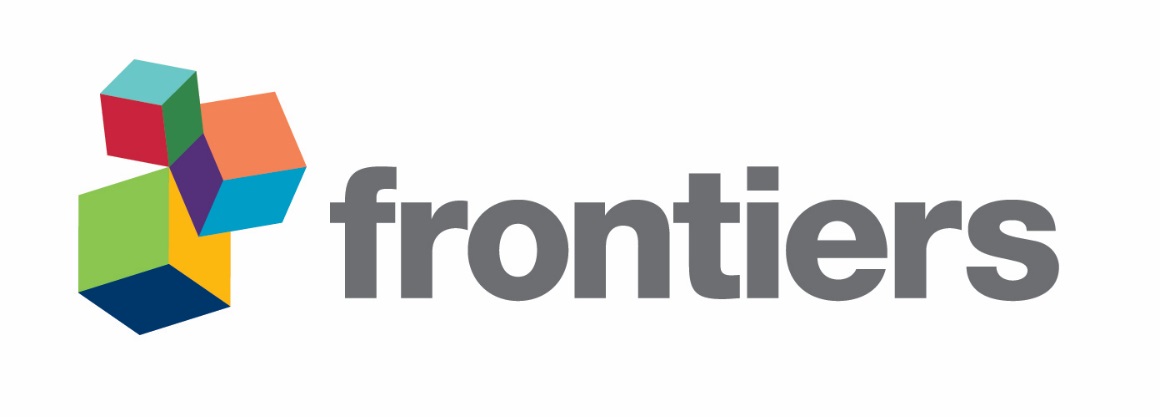
**
